# Kinematics Analysis of Cervical Rotation-Traction Manipulation Measured by a Motion Capture System

**DOI:** 10.1155/2017/5293916

**Published:** 2017-10-11

**Authors:** Zhu Liguo, Feng Minshan, Yin Xunlu, Wang Shangquan, Yu Jie

**Affiliations:** Department of Spine, Wangjing Hospital, China Academy of Chinese Medical Sciences, Beijing 100102, China

## Abstract

**Objectives:**

To analyze the kinematics of cervical rotation-traction manipulation (CRTM).

**Methods:**

An experimental study measuring the kinematics of CRTM was conducted. A total of 18 healthy volunteers participated in the study. A single manipulator operated the CRTM for all subjects. Motion capture technology was adopted to track the trajectory during the CRTM operation.

**Results:**

The manipulated side did not influence the cervical spine motion. The motion ranges obtained during CRTM were well below the active range of motion reported in the literature. The head rotation angle after thrusting was less than the angle of the rotary-position (*P* < 0.05). There was no significant difference in the head rotation angle between pretraction and upward-thrust. The thrust direction of CRTM was mainly upward. The thrust operation was of high-velocity and low-amplitude (thrust velocity: 203.06 ± 49.95 mm/s; thrust acceleration: 3836.27 ± 1262.28 mm/s^2^; thrust displacement: 3.25 ± 1.30 mm).

**Conclusions:**

CRTM has clear operation steps and repeatability that is suitable for clinical application.

## 1. Introduction

Cervical rotational manipulation (CRM) is one of the effective treatments for cervical spondylosis [1, 2]. There are many kinds of CRM around the world. CRM has a long history of use in China. Spinal manipulation was used medicinally as early as 2700 BC in ancient Chinese medicine. At present, the CRM techniques used clinically in China can be mainly divided into unfixed point rotational manipulation and fixed point rotational manipulation [3]. A representative of the former is Sun's CRM [4], while a representative of the latter is Feng's CRM [5]. The two kinds of manipulations have their own characteristics. Unfixed point rotational manipulation has a wider action range and better effects for the upper cervical spine, while fixed point rotational manipulation has a limited action range and is adopted for the lower cervical spine [6].

Both kinds of CRM are skillful treatments that depend on doctors' experience. Beginners often find it difficult to grasp the crucial operation of CRM within a short time. Consequently, they need to spend a long time performing repeated operations and explorations in practice, and the associated learning efficiency is low. Furthermore, some risks might be associated with CRM. Excessive force, excessive amplitude in one or several directions, or a combination of these parameters can cause stroke, atlantoaxial dislocation, vertebral artery spasm, nausea, vomiting, and so on [7–12]. To some extent, the risks of CRM are associated with the competency and skills of the practitioners; what is more, current data are often based on retrospective case-control data with selection and information bias often present, meaning that risks may be underrepresented/underreported [13]. These reasons limit the promotion of CRM in China.

Therefore, we have improved the operating method for CRM from the basic procedure for Sun's CRM and named it “cervical rotation-traction manipulation” (CRTM). The most prominent feature of CRTM is that it decomposes the core operations of rotary-thrust into three steps: subject's active rotary-position, operator's pretraction, and operator's upward-thrust. CRTM has a clear procedure for operation that is easy to grasp. Study shows that CRTM can reduce the risk of excessive rotation through the subject's active rotary-position and the operator's upward-thrust [14]. The cervical ROM improvement of CRTM for cervical spondylosis radiculopathy has been demonstrated in a multicenter randomized controlled clinical study [15, 16]. Currently, CRTM is classified as one of the “one hundred Chinese clinical utility extension projects” of the State Administration of Traditional Chinese Medicine.

It is meaningful to quantitatively analyze the kinematics of spinal manipulative therapy [17]. Since 1997, basic science white papers have recommended the identification and measurement of critical biomechanical parameters in a manipulation [18, 19]. This can clearly display the manipulation procedure, identify the mechanical mechanism of manipulation, and be an objective method for training by kinematics analysis.

Triano and Schultz [20] monitored triaxial linear and angular displacement of the head and thorax during cervical manipulative therapy at C2 and compared the values between manipulators using an optoelectronic measurement system. However, they did not investigate the thrust velocities and acceleration. Klein et al. [21] measured the motion and amplitudes obtained during a manipulation known as the “composite levers technique” on two cervical levels (C3 and C5) using a three-dimensional electrogoniometer. The manipulation was operated by a single practitioner on 14 asymptomatic volunteers. The results of the study suggested that for the kind of manipulation applied, the maximal amplitude between the head and the trunk did not exceed the physiological active range of motion. The amplitude for rotation, which is generally assumed to involve the greatest risks for negative side effects, was significantly lower than during active motion. Ngan et al. [22] successfully used motion capture technology to analyze the kinematic parameters of a lower cervical rotational manipulation (Maitland) in 2005. Williams and Cuesta-Vargas [23] used an inertial sensor mounted on the forehead of the subject to measure the cervical kinematics in 13 asymptomatic subjects who received a right-handed and left-handed downslope and upslope manipulation, aimed at C4/5. The results demonstrated differences in the kinematics between the two techniques. Salem and Klein [24] studied 3D kinematics of the cervical spine segments (C4-C5) during premanipulative positioning in vivo.

The above studies showed that it is feasible and effective in analyzing the kinematics of spinal manipulation. The aim of our study was to measure the three-dimensional kinematics of the head relative to the trunk during CRTM and to provide a more detailed understanding of the operating characteristics of CRTM.

## 2. Methods

The cervical spine of 18 volunteers showed that no pathological changes in cervical X-rays joined this study. The subjects comprised 8 females and 10 males aged 22–26 years. A single practicing physiotherapist who specialized in CRTM participated in the study. This study was approved by the Ethics Committee of Wangjing Hospital of China Academy of Chinese Medical Sciences and all subjects signed an informed consent prior to participation.

### 2.1. Instrumentation

The digital motion capture system was composed of 22 digital camera shots (Hawk type, 1.3 million pixels, 250 frame/s, 0.1 mm precision; Motion Analysis Corp., USA), which were taken throughout the room. Cortex Analysis software (developed by Motion Analysis Inc.) was used to analyze and rebuild the three-dimensional images.

### 2.2. CRTM Operation

**Figure 1:**
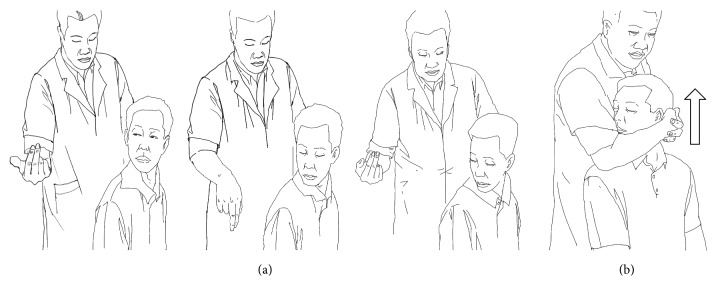
Procedure for CRTM. (a) shows the subject's active rotary-position process of rotation-flexion-rotation, under guidance. (b) shows a physiotherapist performing traction and upward-thrust.

The subjects were manipulated in an upright seated position. The physiotherapists stood behind the subjects. Taking CRTM of the right side as an example (see Figure 1), the parameters were as follows: (1) rotary-position: the patient's head was guided to rotate to the right direction limit, then flexed, and finally rotated to the right direction limit again; (2) pretraction: the patient's mandible was held in the manipulator's forearm and then pulled slowly upward for about 3–5 s; (3) upward-thrust: the head was thrust upward rapidly after pretraction (one or more cracking sounds were always heard when it was successful).

### 2.3. Procedures

**Figure 2:**
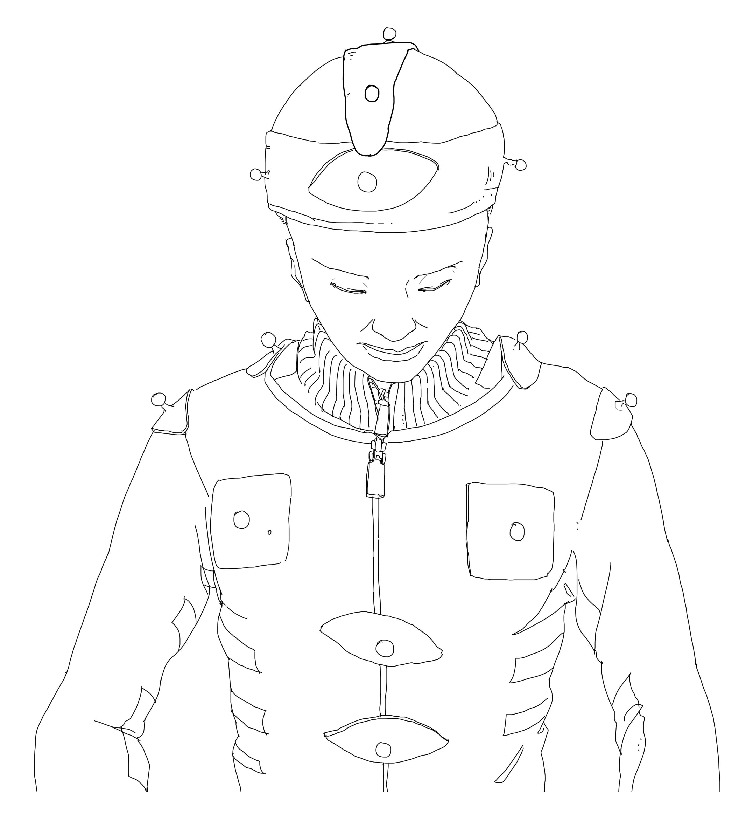
Placement of marker points on the participants.

This study was implemented at Beijing University of Technology. Before the formal experiment, we performed calibration of both the apparatus and the site. A total of 13 marker points were placed on a jacket and hat worn by the participants. The specific placements were as follows (see Figure 2): five marker points on the head (one point each on the bilateral temporal regions, one point on the forehead, one point on the vertex, and one point on the occipital region); four points on the shoulder and neck (one point each on the bilateral acromions, one point each on the midline bilateral clavicles); and four points on the trunk (one point each on the bilateral pectoralis major muscles, one point under the xiphoid, and one point on the upper abdomen).

Each participant stood in the experimental site with open arms to allow completion of the motion capture system calibration before the experiment. When the calibration was finished, the participant was asked to sit, and the doctor operated the CRTM. The motion processes were tracked and saved by the digital motion capture system.

### 2.4. Data Analysis and Postprocessing

**Figure 3:**
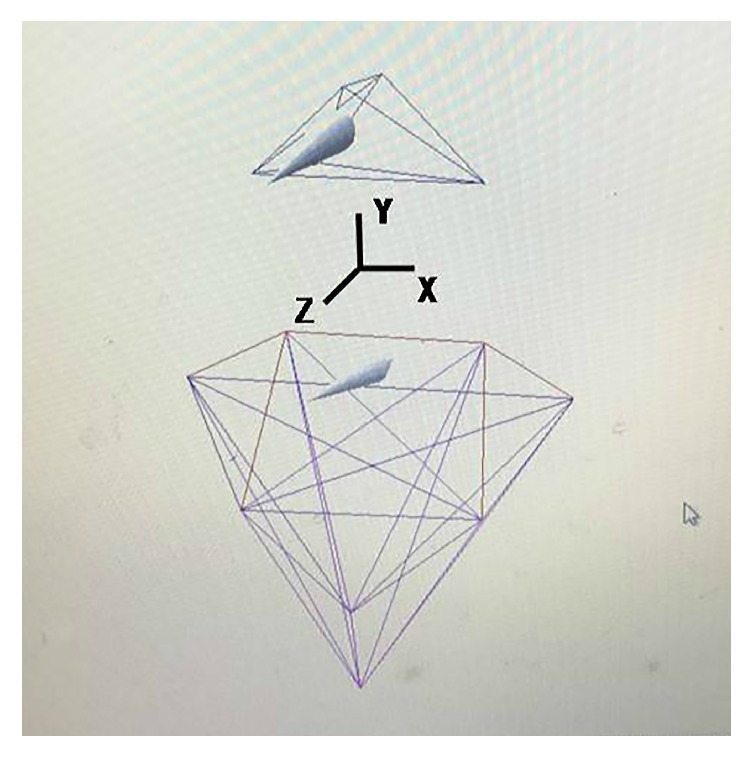
Relationships and coordinate system of the head vector and the trunk vector.

The recorded trajectories of the marker points were postprocessed and analyzed by the Cortex Analysis software program. The marker points and their interrelationships were identified. Next, according to the characteristics of human anatomy, the parts linked to the marker points were bound to establish the head vector and the torso vector. The 3D motion of the head vector was controlled by five marker points on the head. The 3D motion of the torso vector was controlled by four points on the shoulder and neck and four points on the trunk (see Figure 3). Finally, the kinematic parameters of the participant (such as angle, velocity, acceleration, and displacement) were analyzed by the spatial variation of the two vectors. The SPSS version 13.0 statistic package was used for statistical analysis of the data.

## 3. Results

The movement process of the subject's active rotary-position is listed in Table 1. The mean subject active rotary-positions showed no significant difference between left-side and right-side CRTM (*P* > 0.05).

Table 2 presents the neck three-dimensional angles in different states during CRTM. The rotation angles showed significant differences not only between the location state and the pretraction state, but also between the location state and the thrust state (*P* < 0.05). The rotation angles did not differ significantly between the pretraction state and the thrust state or between the bilateral sides in each state (*P* > 0.05).

The mean thrust velocity and thrust acceleration values are shown in Table 3. There were no significant differences in the velocity and acceleration of the *Y*-axis between the bilateral side manipulations (*P* > 0.05).

As shown in Table 4, the thrust displacements showed no significant difference between the bilateral side manipulations (*P* > 0.05).

**Figure 4:**
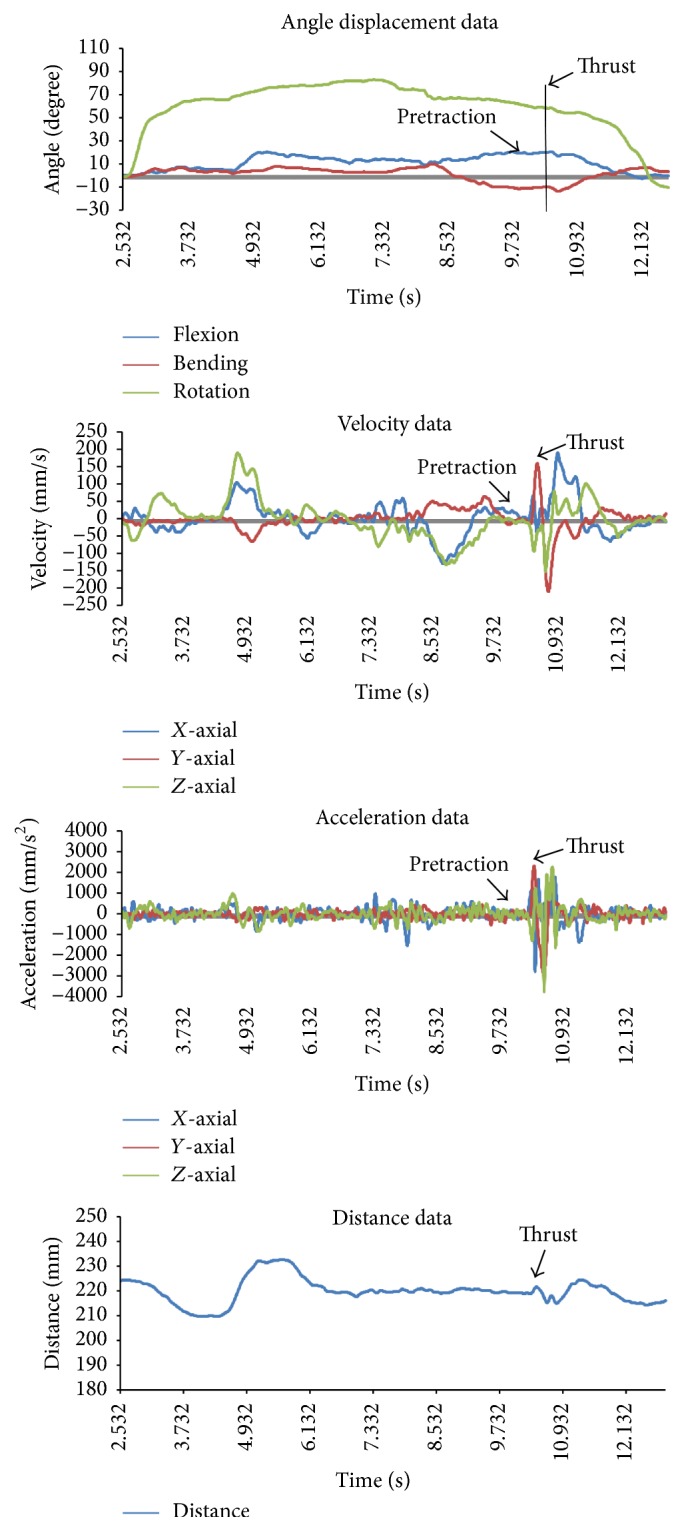
Kinematic pattern for a single participant receiving a cervical rotation-traction manipulation.

A typical kinematic pattern for the CRTM technique is presented in Figure 4.

## 4. Discussion

Motion capture technology has been widely used in many fields, such as the military, 3D animation production, and sports and medicine. Motion capture involves sensing, digitizing, and recording an object in motion. The main advantage of motion capture is the dynamic precision measurement of three-dimensional movement. 3D motion analysis can promote synthetic motion pictures based on human physical or physiological structures with the trajectories of the body motion [26]. Therefore, motion capture technology is suitable for studying the motion of manipulation.

A motion capture system composed of 22 digital camera shots at 250 Hz was used in this study to ensure accuracy. The positions where the marker points are placed must follow the principle of accurate reflection of the skeleton motion and reduce the coverage by the operator during the manipulation operation. Five points were adopted on the head to establish the head vector. The trunk vector was established by eight points, of which four points were on the shoulder. The relative motion between the head vector and the trunk vector is considered to reflect the cervical spine motion.

In this study, the motion trajectories of CRTM were successfully captured. Table 1 shows the head movement process of the rotary-position. The rotary-position was carried out by the subjects: first, rotation to the right direction limit (about 70 degrees); second, flexion (about 30 degrees); and, third, rotation to the right direction limit again (about 10 degrees). When the rotary-position was finished, the neck will be fixed at a position. From the information in Table 1, it can be seen that the process of the rotary-position in CRTM is clear and repeatable. The kinematic characteristics of each step are easy to distinguish. This is helpful for beginners to understand and grasp the process for CRTM.

Table 2 shows the head three-dimensional angles in different states during CRTM. The data in Table 2 showed that the head movement of the participants was in the physiological range of motion during CRTM. The cervical rotation angle of the participant during the thrust phase was less than the angle in the location state, and there was no significant difference in the cervical rotation angles before and after the thrust.

The entire operation of CRTM is broken down into three key steps: subject's active rotary-position; operator's pretraction; and operator's upward-thrust. This can reduce the risk from overrotation by allowing the patient to actively reach the rotary-position. We suggest that the pretraction can open up the facet joints, allowing the intervertebral joint to be more easily mobilized. Moreover, the pretraction before thrust in CRTM can lower the thrust force and range of motion to avoid violent operation.

As shown in Table 3, both the *y*-axis velocity and acceleration were larger than those of the *x*-axis and *z*-axis. This means that the major direction of the thrust is upward. From the data in Tables 3 and 4, we can conclude that the thrust operation of CRTM has the characteristics of high-velocity and low-amplitude, in agreement with previous research [27]. Taking the right-side CRTM as an example, the thrust velocity, acceleration, and displacement were 203.06 + 49.95 mm/s, 3836.27 + 1262.28 mm/s^2^, and 3.25 + 1.30 mm, respectively. We confirm that it is important to grasp this manipulation skill for clinical settings.

## 5. Conclusions

In summary, CRTM has clear steps for operation and is easy to learn and is therefore suitable for clinical application. The thrust skill of high-velocity and low-amplitude should be grasped in clinical settings. This is benefit for the beginners to master the skills of CRTM quickly.

## Supplementary Material

Procedure of CRTM, the related result of CRTM, the related reference.

## Figures and Tables

**Table 1:** Movement process of the active rotary-position (units: angle).

Side	Step 1 rotation	Step 2 flexion	Step 3 rotation again
Right	69.49 (10.17)	27.87 (8.07)	11.03 (5.00)
Left	−72.59 (9.44)	27.10 (6.29)	10.95 (5.90)

*Notes*. Forward flexion, right side rotation, and right lateral flexion are positive.

**Table 2:** Neck three-dimensional angles in different states.

Side	Location state	Pretraction	Thrust	The physiological range [25]
Right				
Flexion	20.7 (9.1)	9.6 (12.3)	8.9 (12.5)	40~60
Rotation	77.2 (9.4)	68.0 (9.9)	69.4 (8.8)	70~90
Bending	1.5 (8.7)	−13.5 (7.7)	−14.9(7.8)	30~45
Left				
Flexion	15.9 (5.8)	6.0 (7.7)	4.8 (8.5)	40~60
Rotation	−81.0 (10.5)	−71.3 (8.4)	−70.7 (8.5)	70~90
Bending	0.1 (10.5)	18.8 (9.3)	21.4 (8.8)	30~45

*Notes*. Values are presented as the mean (standard deviation, SD).

**Table 3:** Thrust velocities and acceleration.

Side	Velocity (mm/s)				Acceleration (mm/s^2^)			
	*X*-axis	*Y*-axis	*Z*-axis	Combined value	*X*-axis	*Y*-axis	*Z*-axis	Combined value
Right	88.21 (52.84)	164.66 (35.64)	−58.70 (46.85)	203.06 (49.95)	1564.13 (908.42)	2780.64 (698.68)	−1406.78 (978.8)	3836.27 (1262.28)
Left	−64.48 (43.01)	181.74 (28.03)	−29.76 (33.22)	196.67 (31.75)	−1146.93 (697.01)	2930.45 (676.92)	−1978.98 (757.7)	3742.4 (803.5)

*Notes*. Values are presented as the mean (SD). The Cartesian coordinate system was adopted to define the three-dimensional axis.

**Table 4:** Thrust displacements (units: mm).

Side	Displacement
Right	3.25 (1.30)
Left	3.14 (1.41)

*Notes*. Values are presented as the mean (SD).
