# Supplementary material for: Kinematics Analysis of Cervical Rotation-Traction Manipulation Measured by a Motion Capture System
Source: Evid Based Complement Alternat Med. 2017 Oct 11;2017:5293916. doi: 10.1155/2017/5293916 (PMC5660796; doi:10.1155/2017/5293916)
Supplement: Supplementary file 1 — Procedure of CRTM, the related result of CRTM, the related reference. [file 5293916.f1.zip › Supplementary Material/Appendix 1.pdf]

·临床研究·

神经根型颈椎病的颈椎活动度的观测及临床意义

朱立国[1] 于杰[1△] 高景华[1] 李金学[1] 李振宇[2] 房敏[3] 李俊杰[4]
张清[1] 王尚全[1] 孙武权[3] 王红东[4] 秦杰[2] 林定坤[2] 陈海云[2]

[摘要] 目的：评价应用颈椎活动仪观测神经根型颈椎病的颈椎活动度方法及其对旋提手法疗效的意义。方法：本组神经根型颈椎病患者 210 例，脱落 12 例，其中试验组 106 例（缺失 5 例），对照组 104 例（缺失 7 例）。试验组采用旋提手法，对照组采用牵引疗法，应用头盔式颈椎活动仪于治疗前和治疗后第 1，3，5，7，9，11，13 天及治疗后 1 个月随访 8 个时间点测量颈椎前屈、后伸、左右侧屈、左右旋转的活动度。结果：两组间颈椎前屈、后伸、左右侧屈、左右旋转的活动度治疗前无差异（$P>0.05$），治疗后均有改善（$P<0.01$，$P<0.05$），而且手法改善颈椎活动优于牵引（$P<0.01$，$P<0.05$）。结论：头盔式颈椎活动仪操作简便，数据准确，对于评价颈椎功能和手法疗效具有重要临床意义。

[关键词] 神经根型颈椎病；颈椎；活动度；手法；正骨
[中图分类号] R681.5+504 [文献标识码] A [文章编号] 1005－0205(2008)01－0001－05

The Measurement for the Range of Motion of Cervical Spine and Clinical Significance

ZHU Liguo[1] YU Jie[1] GAO Jinghua[1] LI Jinxue[1] LI Zhenyu[2] FANG Min[3] LI Junjie[4]
ZHANG Qing[1] WANG Shangquan[1] SUN Wuquan[3] WANG Hongdong[4]
QIN Jie[2] LIN Dingkun[2] CHEN Haiyun[2]
[1]Wangjing Hospital of China Academy of Traditional Chinese Medicine, Beijing 100102, China
[2]Guangdong Provincial Hospital of Traditional Chinese Medicine
[3]Yuyang Hospital Affiliated to Shanghai University of TCM
[4]Hospital of TCM Intergrated with Westtern Medicine, Electic Power Hospital of Beijing

Abstact **Objective**: To evaluate the measurement the range of motion (ROM) of cervical spine and curative effect of rotation-traction manipulation. **Methods**: 210 Patients (12 ablated) with cervicalspondylotic radiculopathy were divided randomly into two groups (the testing group n=101, the control group n=97). The esting group was treated with rotation-traction manipulation, where as the control group was treated with neck traction. The ROMs of cervical spine were measured on pretherapy day 1, 3, 5, 7, 9, 11, 13 and 1 mouth follow-up visit after treatment. **Results**: There was no different on ROM between two groups pretherapy ($P>0.05$). ROMs of cervical spine in two groups were improve ($P<0.01$, $P<0.05$) post-treatment, but the improvement of testing group was better than the control group ($P<0.01$, $P<0.05$). **Conclusion**: The ROM meter is convenient and accur and reliable, which can benefit to evaluate function of cervical spine and curative effect of rotation-traction manipulation.

Key Words Cervicalspondylotic radiculopathy; Cervical spine; Range of motion (ROM); Manipulation; Osteopathtic

神经根型颈椎病患者最为常见的临床表现是颈肩僵痛伴上肢放射痛，多数患者存在颈椎活动受限，并且症状随颈椎的活动而变化或加重。所以颈椎活动度的观测对于病情和疗效的判断，具有重要临床意义，但是目前临床尚无方法可靠，简便易行，适于临床应用的观测方法。

1 资料与方法

1.1 一般资料

本研究入组 210 例患者，脱落 12 例，来自中国中医科学院望京医院、上海中医药大学附属岳阳中西医结合医院、广东省中医院及北京电力医院。试验组 106 例（缺失 5 例），男 31 例，

基金项目：“十五”国家科技攻关课题，课题编号：2004BA721A06
[1] 中国中医科学院望京医院（北京，100102）
[2] 广东省中医院
[3] 上海中医药大学附属中西医结合岳阳医院
[4] 北京电力医院
[△]通讯作者，Tel：010－84739194，E-mail：doctoryujie@yahoo.com.cn

?1994-2015 China Academic Journal Electronic Publishing House. All rights reserved. http://www.cnki.net

女75例,年龄53.59±5.60岁,病程18.40±20.05月;对照组104例(缺失7例),男32例,女72例,年龄52.68±6.12岁,病程19.94±23.87月。两组基数资料比较,P值均大于0.05,两组具有可比性。

1.2 诊断标准

参照1993年全国第二届颈椎病专题座谈会颈椎病的诊断标准[1]:(1)具有典型的根型症状(麻木、疼痛),且范围与颈脊神经支配所区域相一致;(2)压颈试验或臂丛神经牵拉试验阳性;(3)影像学所见与临床表现相符合;(4)痛点封闭无显效(已经明确诊断者可不作此试验);(5)除外颈椎外病变(胸廓综合征、网球肘、腕管综合征、肩周炎等)所致以上肢疼痛为主的疾患。

1.3 纳入标准

(1)综合上述诊断标准;(2)年龄45~65岁;(3)病程小于(或等于)5年;(4)如果已经接受过其他手法或保守治疗,经过5天以上的洗脱期。

1.4 排除标准

(1)合并颈椎肿瘤、结核、骨髓炎及严重骨质疏松症者;(2)可疑脊柱损伤伴脊髓损伤者;(3)发育性椎管狭窄者(椎管比值=椎管矢状径/椎体矢状径< 0.75);(4)患严重的心、肺、脑及血液系统疾病者;(5)治疗部位有严重皮肤损伤或皮肤病者;(5)脊髓型颈椎病等其他类型颈椎病;(6)曾经接受颈椎手术治疗和颈椎畸形者。

1.5 剔除病例标准

(1)试验组连续治疗3次以上脱失的病例,按无效计算;(2)对照组连续治疗4次以上脱失的病例,按有效计算。

1.6 随机与分组

采用中央随机方法,各中心指定2名人员承担数据管理员应用电话或网络由国家药品临床研究基地南京中医药大学附属医院随机分配系统获取并记录申请序号和研究组别,并打印随机分配的电子邮件,转交研究者粘贴在病例观察表上。

1.7 治疗方法

治疗者均为高年主治医师以上职称,经2周的培训考核,保证治疗的正规和统一。

1.7.1 试验组采用旋提手法[2]:(1)病人端坐位,颈部自然放松,医者采用按法、揉法、滚法等手法放松颈部软组织5~10min;(2)让患者的头部主动水平旋转,最大屈曲,再次旋转到最大范围;(3)医生以肘部托患者下颌,轻轻向上牵引3~5s;(4)嘱其放松肌肉,肘部用短力快速向上提拉。操作成功可以听到一声或多声弹响;(5)应用提、拿等手法再次将颈肩部肌肉放松;(6)隔天1次,每次约10~15min。

1.7.2 对照组采用牵引疗法[3]:(1)患者取坐位,带枕颌布兜牵引,头部向前微屈,约前倾10°~15°,并以患者感觉舒适且能减轻症状为准;(2)牵引重量从3kg开始,按0.5 kg标准逐渐增加重量,最大重量不超过6kg;(3)每天1次,每次牵引30 min。

1.7.3 疗程安排 试验组手法治疗持续2周,隔日1次,共7次;对照组牵引治疗方持续2周,每日1次,共14次,症状消失可以停止治疗。于治疗前和治疗第1,3,5,7,9,11,13天及治疗后1个月随访8个时间点进行观察,各中心由2名专职观测者记录观察指标。

1.8 颈椎活动度测量

设备:头盔式颈椎活动仪,国家专利号ZL2006 2 0002019.0,外形呈头盔状,具有开口环形本体,正前方、侧方和顶端设嵌有角度仪的透视板,其中心穿过一个可以自由摆动的指针,测量值精确到1度。各中心由2名专职观测者,经操作培训,并经5次试测,满足测量值稳定性者可进行试验。应用头盔式颈椎活动仪于观测的9个时点测量颈椎活动度:(1)患者抬头挺胸的端坐位,背靠椅背,保持胸椎不动,下颌微收,目光平视前方,头盔前下缘置于眉弓上缘,整个头盔下缘处于同一水平面,三维测量表指针归零,此为标准体位。(2)分别测量前屈、后伸、左右侧屈、左右旋转三维六个方向的活动角度,每个角度患者主动活动极限数值与标准体位指针所指数值之差作为该角度活动度。(3)测量一个轴线位颈椎活动时,要求其他两个轴线活动指针保持不变,以避免各角度间的耦合运动。

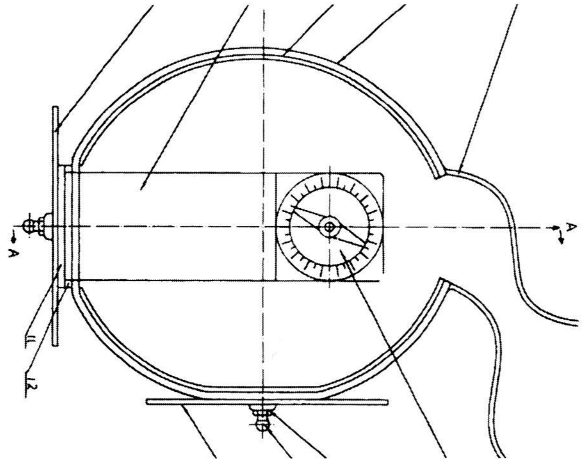

图1 头盔式颈椎活动仪简图

1.9 数据管理和统计方法

完成数据盲态审核报告后,锁定数据库,由国家药品临床研究基地南京中医药大学附属医院进行数据管理和统计分析:(1)采用SAS统计分析软件进行计算;(2)所有的统计检验均采用双侧检验,$P \leqslant 0.05$将被认为所检验的差别有统计意义;(3)各次就诊的计量资料将采用均数±标准差($\bar{x} \pm S$)进行统计描述,组间比较采用t检验,两组治疗前后的差值变化采用Wilcoxon秩和检验。

2 结果

2.1 两组治疗前后颈椎前屈活动度

两组治疗前后颈椎前屈活动度,经t检验,首诊$P > 0.05$,差异无统计学意义;第7次复诊$P < 0.05$,1月随访$P < 0.01$,差异有统计学意义。各次复诊与首诊的差值,经秩和检验,3、4、6次复诊$P < 0.05$,1、6次复诊和1月随访$P < 0.01$,差异有统计学意义,说明手法改善前屈活动优于牵引(表1)。

2.2 两组治疗前后颈椎后伸活动度

两组治疗前后颈椎后伸活动度,经t检验,首诊、第7次复诊$P > 0.05$,差异无统计学意义;1月随访$P < 0.05$,差异有统计学意义;各次复诊与首诊的差值,经秩和检验,$P < 0.01$,差异有统计学意义,说明手法改善后伸活动优于牵引(表2)。

2.3 两组治疗前后颈椎左侧屈活动度

两组治疗前后颈椎左侧屈活动度,经t检验,首诊$P > 0.05$,差异无统计学意义;第7次复诊、1月随访$P < 0.01$,差异有统计学意义。各次复诊与首诊的差值,经秩和检验,4次复诊$P < 0.05$,

?1994-2015 China Academic Journal Electronic Publishing House. All rights reserved. http://www.cnki.net

2、3、5、6、7 次复诊和 1 月随访 $P<0.01$，差异有统计学意义，说明手法改善左侧屈活动优于牵引(表 3)。

2.4　两组治疗前后颈椎右侧屈活动度

两组治疗前后颈椎右侧屈活动度，经 t 检验，首诊 $P>0.05$，差异无统计学意义；第 7 次复诊、1 月随访 $P<0.01$，差异有统计学意义。各次复诊与首诊的差值，经秩和检验，2、3、5 次复诊 $P<0.05$，6、7 次复诊和 1 月随访 $P<0.01$，差异有统计学意义，说明手法改善右侧屈活动优于牵引(表 4)。

2.5　两组治疗前后颈椎左旋活动度

两组治疗前后颈椎左旋活动度，经 t 检验，首诊 $P>0.05$，差异无统计学意义；第 7 次复诊、1 月随访 $P<0.01$，差异有统计学意义。各次复诊与首诊的差值，经秩和检验，5 次复诊 $P<0.05$，其余 $P<0.01$，差异有统计学意义，说明手法改善左旋活动优于牵引(表 5)。

2.6　两组治疗前后颈椎右旋活动度

两组治疗前后颈椎左旋活动度，经 t 检验，首诊 $P>0.05$，差异无统计学意义；第 7 次复诊、1 月随访 $P<0.01$，差异有统计学意义。各次复诊与首诊的差值，经秩和检验，1、2、4 次复诊 $P<0.05$，5、6、7 次复诊和 1 月随访 $P<0.01$，差异有统计学意义，说明手法改善左旋活动优于牵引(表 6)。

表 1　两组治疗前后颈椎前屈活动度　（度）

访　视	试验组	对照组	统计量	P 值
首　诊	41.81±9.83	43.17±10.42	$t=0.97$	$P=0.3310$
7 次复诊	48.52±7.14	46.30±7.62	$t=2.18$	$P=0.0304$
1 月随访	48.81±8.07	46.01±7.32	$t=2.63$	$P=0.0093$
1 次复诊－首诊	2.48±5.30	0.03±5.72	$Z=-4.21$	$P=0.0000$
2 次复诊－首诊	3.57±8.00	1.63±8.52	$Z=-1.69$	$P=0.0915$
3 次复诊－首诊	4.03±8.18	1.39±9.68	$Z=-2.39$	$P=0.0170$
4 次复诊－首诊	4.38±8.13	1.97±9.14	$Z=-2.39$	$P=0.0169$
5 次复诊－首诊	4.68±9.15	2.36±8.45	$Z=-1.93$	$P=0.0539$
6 次复诊－首诊	5.79±9.68	3.08±8.77	$Z=-2.30$	$P=0.0213$
7 次复诊－首诊	6.74±9.98	3.13±8.91	$Z=-3.19$	$P=0.0014$
1 月随访－首诊	7.03±10.47	2.84±9.50	$Z=-3.39$	$P=0.0007$

表 2　两组治疗前后颈椎后伸活动度　（度）

访　视	试验组	对照组	统计量	P 值
首　诊	41.64±11.25	43.31±12.40	$t=1.02$	$P=0.3087$
7次复诊	49.05±7.14	46.73±10.16	校正 $t=1.91$	$P=0.0583$
1月随访	48.52±7.87	46.15±9.30	$t=1.99$	$P=0.0480$
1 次复诊－首诊	2.60±6.37	0.47±6.94	$Z=-2.85$	$P=0.0044$
2 次复诊－首诊	3.10±7.94	0.30±10.12	$Z=-3.25$	$P=0.0012$
3 次复诊－首诊	3.51±8.75	0.47±10.45	$Z=-3.05$	$P=0.0023$
4 次复诊－首诊	3.96±8.75	1.31±11.88	$Z=-2.65$	$P=0.0081$
5 次复诊－首诊	4.94±9.19	2.56±11.51	$Z=-2.65$	$P=0.0081$
6 次复诊－首诊	6.01±8.87	2.75±11.57	$Z=-2.95$	$P=0.0032$
7 次复诊－首诊	7.39±9.36	3.42±12.71	$Z=-3.52$	$P=0.0004$
1 月随访－首诊	6.87±11.32	2.85±11.24	$Z=-3.31$	$P=0.0009$

表 3　两组治疗前后颈椎左侧屈活动度　（度）

访　视	试验组	对照组	统计量	P 值
首　诊	35.99±6.38	36.51±7.42	$t=0.54$	$P=0.5871$
7次复诊	43.94±4.74	40.77±6.22	校正 $t=4.15$	$P=0.0001$
1月随访	44.33±5.37	41.06±5.94	$t=4.18$	$P=0.0000$
1 次复诊－首诊	1.75±5.18	0.56±3.90	$Z=-1.63$	$P=0.1040$
2 次复诊－首诊	2.88±5.62	0.89±5.40	$Z=-2.73$	$P=0.0063$
3 次复诊－首诊	4.38±7.03	1.13±6.35	$Z=-3.48$	$P=0.0005$
4 次复诊－首诊	4.33±6.58	1.86±5.99	$Z=-2.22$	$P=0.0263$
5 次复诊－首诊	5.21±6.72	2.82±7.36	$Z=-2.65$	$P=0.0081$
6 次复诊－首诊	6.79±7.14	3.63±6.94	$Z=-3.07$	$P=0.0022$
7 次复诊－首诊	7.99±7.32	4.26±7.45	$Z=-3.83$	$P=0.0001$

?1994-2015 China Academic Journal Electronic Publishing House. All rights reserved.　http://www.cnki.net

1 月随访—首诊	8.38±7.83	4.55±6.92	Z=－3.83	P=0.0001

表 4 两组治疗前后颈椎右侧屈活动度 （度）

访 视	试验组	对照组	统计量	P 值
首 诊	36.40±6.89	36.48±9.18	校正 t=0.08	P=0.9400
7 次复诊	43.57±5.45	40.56±6.57	t=3.61	P=0.0004
1 月随访	44.00±5.48	40.63±6.45	t=4.08	P=0.0001
1 次复诊—首诊	1.01±5.20	－0.16±4.71	Z=1.47	P=0.1407
2 次复诊—首诊	1.83±5.68	－0.02±6.20	Z=2.17	P=0.0300—
3 次复诊—首诊	3.28±5.97	1.24±7.41	Z=－2.12	P=0.0340
4 次复诊—首诊	3.60±6.19	2.13±7.71	Z=－1.61	P=0.1070
5 次复诊—首诊	4.83±6.51	2.54±8.15	Z=－2.25	P=0.0246
6 次复诊—首诊	6.10±7.38	2.85±7.91	Z=－3.32	P=0.0009
7 次复诊—首诊	7.21±8.13	4.08±8.30	Z=－2.99	P=0.0027
1 月随访—首诊	7.64±8.06	4.14±8.40	Z=－3.81	P=0.0001

表 5 两组治疗前后颈椎左旋活动度 （度）

访 视	试验组	对照组	统计量	P 值
首 诊	61.02±12.26	61.28±12.97	t=0.15	P=0.8815
7 次复诊	73.48±6.69	67.74±8.36	校正 t=5.47	P=0.0000
1 月随访	74.33±7.27	68.27±8.78	t=5.44	P=0.0000
1 次复诊—首诊	2.31±7.87	0.29±7.47	Z=－3.06	P=0.0022
2 次复诊—首诊	3.97±8.80	0.74±9.93	Z=－2.82	P=0.0048
3 次复诊—首诊	5.97±9.87	1.46±9.96	Z=－3.72	P=0.0002
4 次复诊—首诊	7.21±11.03	3.00±11.04	Z=－2.96	P=0.0031
5 次复诊—首诊	8.39±11.16	5.36±11.63	Z=－2.26	P=0.0240
6 次复诊—首诊	11.19±10.95	5.40±11.91	Z=－4.44	P=0.0000
7 次复诊—首诊	12.54±12.21	6.46±12.54	Z=－4.13	P=0.0000
1 月随访—首诊	13.40±12.37	6.99±12.57	Z=－4.21	P=0.0000

表 6 两组治疗前后颈椎右旋活动度 （度）

访 视	试验组	对照组	统计量	P 值
首 诊	62.71±11.26	62.77±13.18	t=0.04	P=0.9720
7 次复诊	73.63±5.82	68.46±7.98	校正 t=5.34	P=0.0000
1 月随访	74.29±6.14	68.61±8.37	t=5.59	P=0.0000
1 次复诊—首诊	2.63±7.73	1.10±9.05	Z=－2.45	P=0.0142
2 次复诊—首诊	3.44±6.89	1.26±8.91	Z=－2.28	P=0.0224
3 次复诊—首诊	4.55±9.45	1.89±10.83	Z=－1.36	P=0.1749
4 次复诊—首诊	5.98±9.36	2.52±11.01	Z=－2.24	P=0.0254
5 次复诊—首诊	7.65±9.87	3.69±11.58	Z=－2.68	P=0.0073
6 次复诊—首诊	9.90±10.98	4.47±12.53	Z=－3.34	P=0.0008
7 次复诊—首诊	11.04±10.98	5.68±13.43	Z=－3.16	P=0.0016
1 月随访—首诊	11.70±10.79	5.83±12.77	Z=－3.98	P=0.0001

3 讨论

3.1 神经根型颈椎病颈椎活动度的病理生理意义

颈椎是脊柱活动度最大的节段，也是病变最常见节段。大多数神经根型颈椎病患者存在不同程度颈椎活动受限，是临床常用的检查项目。

颈椎前屈以 C5/6、C6/7 节段最大，后伸 C4/5 为中心，侧弯以 C2 至 C7 完成，旋转以 C1/2 为主，这对于颈椎病早期诊断、判断病变程度的轻重、颈椎节段功能、定位诊断和病变向愈的趋势和程度具有临床意义。颈椎过屈主要受后纵韧带、黄韧带、棘间韧带、项韧带、颈后肌群限制；颈椎过伸主要受前纵韧带、颈前肌群阻止；颈椎侧弯主要依靠双侧关节囊及附近韧带、肌肉相互制约、协同完成。神经根型颈椎病颈椎活动受限的病理机制尚未见系统研究，可能有肌肉痉挛、滑膜嵌顿、关节脱位和半脱位、小关节的关节囊粘及韧带粘连、挛缩等几方面原因。

?1994-2015 China Academic Journal Electronic Publishing House. All rights reserved. http://www.cnki.net

3.2 颈椎活动度的观测

颈椎活动度的个体差异较大, 加之与年龄、体型及锻炼情况关系密切, 加之测量方法不尽相同, 各家报告的差异较大。一般正常活动度: 颈椎中立位屈伸活动度为 35°~45°, 左右侧弯为 45°, 左右旋转为 60°~80°。陈肇辉等[4]应用摄像机以及图像处理系统测量 10 名无颈椎疾患自愿受试者(平均年龄 24.80±3.97)颈椎主动活动度, 前屈 47.3±2.9, 后伸 38.7±3.4, 左侧屈 41.9±2.2, 右侧屈 42.9±1.7, 左旋 68.3±6.3, 右旋 68.5±5.6。本研究中患者首诊时各角度活动范围均有较为明显受限, 治疗后(如第 7 次复诊)均趋于正常。

目前, 临床常用方法尚有不足, 如目测法[5]存在不准确性, 受前一次干扰造成下一次很难避免的误差, 敏感性差; 皮尺测量法[6], 以骨性标志定位, 简便廉价, 旋转和侧弯运动可靠性较好, 但证据较为混杂, 其可靠性、可行性尚需证实; 国外报道的颈椎活动测量仪优于一般测量仪和目测, 但是应用不容易, 中立位测量可靠性差[7], 不同测量者可靠性低[8]。

本研究中的头盔式颈椎活动仪操作简便, 适于临床应用, 可分别测量颈椎三维六个方向的活动, 并且观测其中一维活动时, 注意保持其他两维指针不动, 从而避免耦合运动。另外注意患者于标准体位, 活动中保持胸椎不动。在同一测量者的不同测量中和不同测量者中稳定性较好。

颈椎整体测量应与节段测量(需影像学测量)相结合, 不同年龄、性别、身高、体型等人群的区别, 尚有待于进一步研究。

3.3 旋提手法对颈椎病活动度的改善

一般将手法治疗神经根型颈椎病的机理归纳为缓解肌肉痉挛, 纠正关节错位, 松解粘连, 解除滑膜嵌顿, 对颈椎椎间盘粘弹性与应力发布的调整等[9]几方面, 从而恢复颈、头部的生物力学的生理性平衡状态, 阻止或减缓颈椎病变恶性循环, 使颈肩肌的病变、韧带的病变及颈椎生理曲度的病变得到改善和康复, 从而达到消除症状, 治愈疾病的目的, 而能完成颈椎的正常功能。而且, 本研究规范的旋提手法将患者主动旋转与医生上提分离, 使手法易于掌握, 增加可控性和安全性。本手法缓解肌肉痉挛, 松解局部粘连, 改善关节囊、韧带的挛缩, 滑利关节, 从而恢复颈椎活动范围。

本研究中通过颈椎活动仪测量颈椎活动度, 为评价手法治疗神经根型颈椎病的疗效提供量化依据。第 1 次复诊手法组颈椎前屈、后伸、左旋、右旋改善优于牵引组, 说明手法起效较快; 治疗后期和随访期间活动度均优于牵引组, 说明手法作用较为持久, 随访结果说明手法打破病变的恶性循环, 恢复颈、头部的生物力学的生理性平衡状态, 为长久的自身向愈建立基础。

参考文献

[1] 孙宇. 第二届颈椎病专题座谈会纪要[C]. 中华外科杂志, 1993, 31(8): 472-476.

[2] 朱立国, 张清, 高景华, 等. 旋转手法治疗神经根型颈椎病的临床疗效研究[J]. 中国骨伤, 2005, 18(8): 489-490.

[3] 杨克勤. 脊柱疾患的临床与研究[M]. 北京: 北京出版社, 1993: 539.

[4] 陈肇辉, 黄文化, 赵卫东, 等. 人体颈部活动度在体测量方法研究[J]. 中国临床解剖学杂志, 2003, 21(3): 411-412.

[5] VIIKARE-JUNTURA E. Interexaminer reliability of observations in physical examinations of the neck[J]. Phys Ther, 1987, 67: 1526-1532.

[6] VIANEN J V, KOKKO M L, HEIKKILA S, et al. Neck mobility assessment in ankyloseing spondylitis: aclnical study of nine measurements including new tape methods for cervical rotation and lateral flexion[J]. Br J Rheumatol, 1998, 37: 377-381.

[7] NILSSON N, Measuring passive cervical motion: a study of reliability[J]. J Manipulative Physiol Ther, 1995, 18: 293-297.

[8] NILSSON N, CHRISTINSEN H W, HARTIGSEN J. The interexaminer reliability of measuring passive cervical range of motion, revisted[J]. J Manipulative Physiol Ther, 1996, 19: 302-305.

[9] 朱立国, 于杰, 高景华. 旋转手法治疗神经根型颈椎病研究概述[J]. 中医正骨, 2005, 17(8): 69-70.

(收稿日期: 2007—05—19)

全国首家内病外治经筋新疗法培训学校常年招生

王卫平医师出身中医世家, 以祖传心法为基础, 结合现代医学, 研究成功经筋新疗法, 该疗法是教科书上没有创新理念、创新技术, 具有适应症广, 标本兼治, "简、便、验、廉"的特点, 不吃药, 对肝肾无损害, 通过手法, 药磁灸, 解肌穴注经筋新穴, 治疗慢性病有特效, 特别对颈肩腰腿痛有立竿见影的功能, 可当面止疼, 常规疗法无法治疗的病痛在这里很快康复, 学员先考察是否属实, 再决定学习, 不符实报销路费, 免去你花钱学不到技术的后顾之忧, 残疾人减免学费。

鼻炎头痛学习班重点讲解为什么鼻炎, 鼻窦炎不在鼻部、肺部治疗能神奇治愈, 顽固性正偏头痛、头晕、三叉神经痛、高血压、失眠不在头部治疗的新理论和快速治愈方法。

类风湿颈肩腰痛痛学习班, 重点讲解为什么腰间盘突出不治腰, 腿痛不治腿, 膝关节炎、增生不治关节, 足跟痛不治足, 肩周炎有真假, 骨质增生能软化, 治股骨头坏死与膝关节炎取穴一样, 等全身关节软组织痛的新理论和快速治愈方法。

内科病学习班讲解Ⅱ型糖尿病、胃病、结肠炎、冠心病、胆囊炎、胆结石、小儿腹泻、厌食同治一个病根的新理论和快速治愈方法。

创新理论, 创新技术, 提升你的诊疗水平!

每月 1 日、15 日开班, 学期 5 天, 面授、函授均可, 经筋 7 星磁针灸专利号 0720139708.0, 经筋药灸专利号 0710084831.1

通讯地址: 河北省巨鹿县内病外治经筋新疗法培训学校, 巨鹿县健康路老县医院对过

联 系 人: 王卫平 电话: 0319—4311158 手机: 13930962015(限发短信) 邮编: 055250

农行卡号: 6228481250018026419 邮政储蓄帐号: 601331001200114327

?1994-2015 China Academic Journal Electronic Publishing House. All rights reserved. http://www.cnki.net
