# Supplementary material for: Kinematics Analysis of Cervical Rotation-Traction Manipulation Measured by a Motion Capture System
Source: Evid Based Complement Alternat Med. 2017 Oct 11;2017:5293916. doi: 10.1155/2017/5293916 (PMC5660796; doi:10.1155/2017/5293916)
Supplement: Supplementary file 1 — Procedure of CRTM, the related result of CRTM, the related reference. [file 5293916.f1.zip › Supplementary Material/Appendix 2.pdf]

旋转手法治疗神经根型颈椎病研究概述

中国中医研究院望京医院（北京　100102）
朱立国　于　杰　高景华

随着社会发展，颈椎病已经成为骨科发病率之首。神经根型在颈椎病发病中占 50%～60%，因颈椎椎间盘退行性改变及其继发性病理改变累计神经根为主，并出现与病变阶段相一致的根性症状和体征[1]。非手术疗法是神经根型颈椎病主要疗法，其中手法治疗因其疗效肯定、见效快、副作用低等原因而广泛应用于临床。旋转手法是治疗颈椎病的关键手法，现就旋转手法治疗神经根型颈椎病研究概述如下。

1　旋转手法治疗神经根型颈椎病操作

1.1　操作要领　旋转手法不是孤立的手法，它是一系列手法中最重要的组成部分，在颈椎病治疗一般采取三步手法[2]。第 1 步是准备手法（点、按、揉、捻、滚等法）：放松痉挛僵硬的颈肩肌群，促进局部血液循环，达到舒筋通络，宣通气血，解痉镇痛的效果，同时也为下一步手法的运用打好基础。第 2 步是旋转复位手法：是治疗颈椎病重点手法，临床常用、教科书介绍较多的颈部旋转手法主要有定点旋转手法和不定点旋转手法两种。①定点旋转手法：患者端坐位，颈部自然放松，术者立于患者身后，双手触诊检查偏歪的棘突，然后患者向棘突偏歪侧主动旋转至最大限度。术者一手拇指顶推偏歪的棘突，其余四指扶持颈部，另手前臂掌面紧贴下颌，掌心抱住后枕部。将抱头的手向上牵拉并向棘突偏歪侧旋转头颈部，同时推顶棘突的拇指突然发力推顶棘突。此时多可听到一声“咔嗒”声，拇指下的棘突可有轻度位移。②不定点旋转手法：患者端坐位，术者立于患者身后。用右手或右前臂置于患者颌下，左手托住枕部，轻提并且做颈部旋转运动 2～3 次。使患者颈部肌肉放松，然后上提，牵引颈部，并使其保持中立位，牵引的同时将患者的头颈右旋至有固定感时，右手或右前臂快速发力旋转颈部，此时即可听到一连串的弹响声。之后以同样手法向左侧旋转复位 1 次[2]。目前，临床应用的颈椎旋转手法繁多，但离不开旋转与推扳，多可归入此两大类之中。第 3 步为善后手法（提、拿、劈、散、归合等法）：放松颈肩部肌群，进一步解除肌肉痉挛，改善血液循环，增加局部血液供应，消除软组织的炎性反应，从而起到疏风通络、消炎止痛、调和气血之功。

1.2　体位与角度　旋转手法常用体位有坐位、卧位两种，由于坐位简便、易行、角度易于调控、牵引方向与重力相反等优点为临床常用；郑秀华等[3,4]认为卧位减轻了患者坐姿的恐惧和紧张而产生的颈肌对抗，由坐而改卧位使颈肌及全身肌肉更为放松，给手法实施消除了负面影响，为旋转复位的成功操作奠定了基础，同时颈肌放松，使手法更加轻巧灵便，得心应手，安全稳妥。同时，避免了过度、勉强用力所造成的损伤，方便对手法过程出现眩晕等问题的应急处理。

患者颈椎前屈 15～20° 时黄韧带、后纵韧带纤维被拉长，可使颈椎椎管矢径和神经根管（椎间孔）相应增大，拔伸时颈部软组织处于紧张状态，利于自身保护机能，增加了颈椎的稳定性。颈椎后伸位时，承重点落在椎体后缘上，$C_{1\sim2}$小关节面接近水平位，灵活性最大，也最不稳，而 $C_{3\sim7}$小关节形成嵌插而相对稳定。因此，手法整复 $C_{1\sim2}$取患者点头位或略后仰位，$C_{3\sim5}$取自然低头位 20～30° 左右，$C_{6\sim7}$取尽量屈颈低头位 30～45° 为宜[5,6]。

1.3　成功标志　临床常将旋转手法后“咔嗒”弹响声作为手法整复成功标志。“咔嗒”弹响与术者所使用的手法、力量以及患者本身的解剖结构等因素均有一定的关系。有学者认为是两个关节面软骨之间的轻快磨擦所致，也有学者认为是嵌顿的滑膜复位造成的，多数学者认为是在快速的旋转过程中关节腔内气体扩散波的震动声，目前尚无共识[7]。从力学观点来分析，做脊柱推拿手法时要使脊柱关节产生“咔嗒”声必须具备依据力，既杠杆力。推拿时脊柱关节出现“咔嗒”声，可以说明推拿的旋转力已经作用到脊柱关节，使其产生了活动，并且使被旋转的关节处于关节运动的极限状态[8]。临床观察出现弹响后患者较未出现者有轻快舒适感，且病变重者弹响声多深闷、滞涩，健康者多轻快、干脆，患者伴随病变向愈，弹响声多由闷涩转变为轻快干脆。反之，则提示疾病逐渐向深发展。但是，操作时不可强求弹响声，反复强扳，应弹响声结合手下有骨关节滑动感为成功主要标准。

2　旋转手法治疗神经根型颈椎病作用机理

目前，旋转手法治疗颈椎病基础研究滞后于临床，机理研究较少，多为推测，有待进一步加强。现有以下几种认识。

2.1　解除肌肉痉挛　骨骼肌张力的异常升高以及肌肉痉挛时，肌肉的形态结构、组织性质、解剖位置和生化等方面并无病理改变，只是功能上出现非协调性的异常收缩。在临床触诊时可以摸到收缩变硬的肌肉或僵硬无弹性的条索状肌腹。除准备手法（点、按、揉、捻、滚等法）松解痉挛僵硬的颈肩肌群，快速推扳和旋转也可突然牵拉松解肌肉的高张力，使异常的肌肉张力恢复正常[9]。

2.2　纠正关节错位　颈椎关节的位置异常致使椎间孔变小和横突孔狭窄扭转位移，刺激、压迫神经根，引发神经根受损的症状。旋转手法可调整椎间盘与神经根的位置、调整钩椎关节、恢复正常的颈椎关节解剖序列，有利于组织水肿的消退、静脉的回流、神经根周围炎症的减退，达到治疗目的。

姜宏[10]通过尸体颈椎标本建立正常与损伤颈椎（$C_{5\sim6}$椎间盘部分切除）力学模型，对比观察整骨手法对颈椎生物力学的影响，结果发现，手法后椎体、椎间盘、小关节及项韧带的应变（应力）均有不同程度的下降，而刚度则呈现不同程度的上升，从而调整了颈椎的静力性平衡，增强了颈部的稳定性。损伤椎施行旋转手法后，其椎体应变较正常颈椎多增加 70%。孙树椿等[11]采用二方向定位摄影术观察模拟旋转手法对已

?1994-2014 China Academic Journal Electronic Publishing House. All rights reserved.　http://www.cnki.net

造成 $C_{3\sim4}$ 和 $C_{4\sim5}$ 椎间小关节紊乱的颈椎标本的作用过程，维持纵向牵引力的同时用力旋转，使处于交锁状态的各椎间、横突关节角与小关节突之间的骨质接触被纵向牵引力拉开，在椎间盘与韧带的牵拉下，尤其是有小关节紊乱的椎间，受扭转的椎间盘和牵拉的韧带更为紧张，被牵拉的椎体向受牵拉方向相反的方向运动，使紊乱的小关节得到调整，紧张的椎间盘、韧带等软组织得到放松。模拟旋转手法主要作用于 $C_{3\sim5}$ 有小关节紊乱的椎体，移位远大于其他无椎间小关节紊乱的椎间活动度。

2.3 松解黏连 Triano J 等[12]研究认为，钩椎关节、小关节、神经根周围以及颈椎管内的某些黏连是造成临床症状的原因之一。颈神经根的肿胀黏连促使椎间孔狭小，引发神经症状。关节周围的软组织黏连，致使关节活动受限和疼痛。颈椎管是一个可扭曲的有效套管，以容纳脊髓。由于各椎体及椎弓均由韧带连接，故椎管是可动的，这就为颈椎旋转手法的操作提供了解剖学依据。旋转手法可使神经根和关节周围的黏连得到一定程度的松解。旋转手法时一般伴有颈椎的侧弯，有研究在新鲜尸体上直接观察颈椎侧弯时椎管结构发生的变化。旋转手法对颈椎管内解剖结构的改变，如椎管截面积、椎管矢状径、神经根袖等具有较明显的作用。旋转时，对侧神经根袖位移明显，这有助于解除神经根袖处的某些黏连。侧弯时，对侧神经根袖位移较明显。前屈旋转对下位神经根位移的影响较大，有利于松解神经根袖处和椎管内的某些黏连，从而达到治疗目的。

2.4 解除滑膜嵌顿 欧洲脊柱推拿治疗者提出，脊柱小关节间的滑膜嵌入是造成脊柱活动受限和疼痛的主要原因[13]。脊柱解剖学研究认为，脊柱小关节内的半月板样结构是颈、腰椎小关节的解剖学特征，该结构受压很可能造成颈痛或反射性肌肉痉挛[14]。因为椎间小关节有独立的关节囊，关节囊内滑膜皱襞上有丰富的感觉神经纤维和 P 物质，当颈随头作各个方向的运动，椎间关节间隙增大时，关节囊内层的滑膜或滑膜皱襞就有可能嵌入，成为疼痛源。颈部的推扳或旋转手法可使嵌入的滑膜或滑膜皱襞得到解除，从而缓解疼痛。但实验未能证明推拿能改变小关节的咬合状态。有人认为旋转手法中同侧关节突上下关节面远离并作切面旋转运动，关节突张开，而对侧关节面靠紧，有利于关节复位，解除嵌顿滑膜及半月板受压，并松解经根管内容和小关节黏连，消除不良刺激，缓解疼痛，此类急性颈痛在脊柱推拿后立即缓解。对脊柱结构神经支配的研究，有助于明确脊柱源性疼痛的周围神经解剖学，对改善临床的手法治疗有益。

2.5 调整颈椎椎间盘黏弹性与应力发布 椎间盘是颈椎承载系统中最为关健的部分，椎间盘的宏观力学行为具有黏弹性。实验研究表明[16]椎间盘的蠕变和髓核以及纤维环所受应力的重新分布有关。其过程不仅包含着结构的变形，而且也包含了液体的自由交换，水分子向髓核中运动对椎间盘黏弹性的维持具有重要意义。姜宏[17]在研究中发现旋转手法可使椎间盘蠕变速度降低 7%～13%，平衡时间延长 5 分钟，应力松弛率降低 10%，载荷平均下降 57%，即产生与椎间盘退变逆向变化的。旋转手法可降低颈椎间盘的蠕变与应力松弛速率，调整颈椎间盘的黏弹性与应力分布，对颈椎间盘的流变学特性起有一定的调整作用，证实手法可提高颈椎的稳定性。另外，损伤的颈椎椎间盘在手法中所承受的应变变化幅度与载荷远远大于正常颈椎，力学上异常受载是椎间盘退变的重要原因。同时研究证明椎间盘蠕变趋向平衡的时间一般为 10～15 分钟左右，并达到饱和，随后即使时间再增加很多，应变也不会再增大。为适应椎间盘的这种黏、弹性规律，临床上理筋手法时间亦可掌握在 15 分钟左右。

3 旋转手法治疗神经根型颈椎病的安全性

颈椎病的手法治疗只要方法正确，操作恰当，严格地选择适应症与禁忌症，不但疗效显著，而且也是安全可靠的。美国统计，每周 120 名患者行推拿治疗，其中 30% 行颈椎旋转手法，比较服用非甾体类抗炎制剂治疗颈痛的危险性是颈部推拿的 100～400 倍[18]。但由于颈椎解剖位置特殊，手法不当引起严重并发症国内外时有报道，手法不当引起的并发症主要有高位脊髓损伤、椎动脉血栓形成、寰枢椎脱位、下关节突骨折及颈椎间盘突出等，主要症状为面色苍白、大汗淋漓、恶心呕吐、头痛眩晕、上肢麻木疼痛加重、颈部活动受限、下肢运动障碍等[19]。以往多考虑手法造成的骨折脱垃和脊髓损伤等，而目前认为最多见的是椎-基底动脉的损伤，极度旋转对血流速度减缓有显著性意义。血流速度高度减缓，意味着颈部供血量急剧下降，是酿成手法操作频发事故的重要原因[19]。

因此，旋转手法操作前需注意几点[20,21]：①明确诊断，摄颈椎 X 线片以排除骨质破坏性病变如肿瘤、结核；②严格掌握适应症与禁忌症，对颈椎间隙感染、椎体骨髓炎、颈椎先天性畸形、椎体骨桥形成、发育性颈椎管狭窄和脊髓型颈椎病等禁用手法治疗；③对伴有严重心血管机能不全、老年人高血压、动脉硬化、心律失常、椎动脉有明显解剖学变异的颈椎病患者慎用手法治疗；④在施行旋转手法时，宜在患者正常的被动活动范围结束时增大活动度，手法加力的特点是轻快、短促，随收随发，使颈部活动在力的推动下超过一般生理限度，但绝不能超越解剖极限。沈国权和严隽陶教授提出对中国脊柱推拿手法的基本要求就是其可控性。所谓的可控性就是对所要推拿节段、被推拿脊柱被动运动幅度及脊柱推拿力量的可控性。

施行旋转复位法时医者必须熟悉局部解剖结构，能够掌握好颈部屈伸、侧屈、头颈沿矢状轴旋转的角度和力度，还必须依据患椎部位的高低、颈椎生理曲线变化、椎间隙大小、颈肌紧张程度而决定采取适宜的角度进行复位。中立位或轻度前屈位牵引颈椎间连接关系改变较少，稳定程度高。操作时不可强求弹响声，应以弹响声和手下有骨关节滑动感为成功主要标准。

旋转手法调整关节，松解黏连，同时必然造成局部组织的轻度损伤，由一个不平衡状态建立一个新的平衡状态，所以临床一般 1 周调整 2～3 次，每次调整完后要求患者当天减少颈部活动，给新的平衡状态的建立和组织的修复一定的时间和机会，且不宜长期、反复施行，尤其是粗暴手法。

4 结　语

目前，包括颈椎旋转手法在内的中医手法流派纷繁，操作方法多种多样，手法操作主要取决于医生个人（下转第 73 页）

?1994-2014 China Academic Journal Electronic Publishing House. All rights reserved. http://www.cnki.net

松动等引发的手术失败病历中，无 1 例与假体的材料质量和生物性能有关，一般认为与以下因素有关：①病人适应症选择不当；②假体的选择不当；③手术者的手术熟练程度和经验的积累。人工腰椎间盘置换与四肢关节置换相比，技术性能要求更高，随着技术的熟练，并发症也将会降低。因此严格地掌握适应症和禁忌症是确保手术成功的前提，熟练而精良的技术是手术成功的保证。加之人工椎间盘的材料和固定技术的不断改进，以及来自于基础科学的椎间盘组织和组织工程学新的进步，随着临床应用成功率的提高，人工椎间盘将会代替椎间融合术而达到更好的临床效果。

4　小　结

综上所述，人工椎间盘是一项新技术，是现代脊柱外科的新概念，可成为脊柱融合术的一种重要替代方法，有广阔的应用发展前景。就目前的文献报道来看，虽然临床满意率尚低于技术成熟的椎间融合术，且对手术的操作技术要求较高，存在潜在的并发症，经济成本高昂，但是对减少患者住院时间，恢复椎间高度，保留椎节节段活动，避免后柱快速退变带来的一系列问题是非常有益的。随着人工椎间盘性能和临床技术的不断改进，人工椎间盘置换术一定能日臻完善，造福人类。

5　参考文献

1　Panjabi MM. Krag MH, Chung TQ, Effects of disc injury on me-chanicalbehacior of the human spine. Spine　1984; 9(7): 707
2　李康华，王华，雷光华，等。腰椎间盘切除与人工椎间盘对关节突关节内压力的影响。中国临床康复　2003; 7(4): 558
3　侯铁胜，屠开元，徐印坎，等。椎间盘切除对腰椎运动学影响的实验研究。中华外科杂志　1989; 27(2): 88
4　戴力扬，屠开元，徐印坎，等。椎间盘切除对腰椎应力分布影响的研究。中华外科杂志　1991; 29(2): 126
5　Cinotti G, David T. Postacchini F, Results of disc prosthesis fater aminimun follow upperiod of 2 years, Spine　1996; 21(8): 995
6　Zeegers WB, bohnen IM, laaper M, et al. artificial disc replacement with the modular type SB Charite Ⅲ. Eur spine J　1999; 8(3): 210
7　杨述华，李进，等。腰椎间盘突出症人工椎间盘置换手术及效果。中国矫形外科杂志　2003; 11(8): 509
8　Link HD, history, design and biomechanics of the LINK SB Charite artificial disc. Eur Spine J　2002; 11(S2): S98
9　Blumenthal SL, Ohnmeiss DD, Guyer R, et al. Artificial intervertebral discs and beyond; a North Americal Spine Society Annual Meeting sympopsium. Spine J　2002; 2(6): 460
10　Lee CK, Langrana NA, Parsons JR, et al. Development of aprothesis intervertebral disc. Spine J　1991; 26(s6): 253
11　黄东生，郝松要，刘尚礼，等。新型人工椎间盘的研制及其静态力学性能。中山医科大学学报　1998; 19: 127
12　Enter P, Steffee A, Mcnillin C, et al. Artificial disc replacement preliminary report with a 3 year minimun follow up. Spine　1993; 18: 1061
13　Main JA, Wells ME, Keller TS, Verterbral prosthesis. USA, A61F002 44. US, patent, 4932975, 1982-01-02
14　Zippel H, "Charite modular" concept, experience and results, in Brock M, Mayer HM, Weigel K(eds); The Artificial Disc, Berlin: Springer Veriag, 1991; pp69～77
15　Enker P, Steffee AD, Total disc replacement, In; Bridwell KH, Fe Wald RL, eds, The Textbook of Spinal Surgery, Second Edition, Philadelphia; Lippincott Raven Publishers　1997; 2275～2288
16　Hochschuler SH, Artificial discs. Proceeding of the 68th AAOS Annual Meeting, San Francisco　2001; 261
17　Griffith SL, Shelokov AP, Vuttner-Janz K, et al. A multicenter retrospective study of the clinical result of LINK SB Charite invertebral prosthesis; the initial European experience. spine　1994; 19: 1842
18　Zeegers WB, Bohnen IM, Laaper M, et al. Artificial disc replacement with the modular type SB Charite Ⅲ. EurSpine J　1999; 8(3): 210
19　Zigler JE, Lumbar disc replacement; is time to stop fusion. AASO Proceedings　2002; 3: 229
20　Eijkelkamp MF, Van Donkeraar CC, Veldhuizen AG, et al. Requirements for on artificial intervertebral disc. Jartiforgans　2001; 24(5): 311
21　McAfee PC, Fedder II, Saiedy S, et al. SB Charite disc replacement; report of 60 prospective randomized cases in a US center. J Spinal Disord Tech　2003; 16(4): 424。

（上接第 70 页）经验和习惯，缺少客观的科学分析与比较。一个角度反映中医手法的繁荣，另一个角度反映手法急需统一规范化、科学化研究（机理、适应症、禁忌症、操作方法等），从而进一步提高手法治疗颈椎病的疗效。

5　参考文献

1　孙宇，陈琪福。第二届颈椎病专题座谈会纪要。中华外科杂志，1993; 31(6): 472
2　张军。孙树椿主任医师治疗颈椎病的手法经验。中国中医骨伤科杂志　2000; 8(4): 60
3　郑秀华，骆运，庞国军。推拿治疗神经根型颈椎病 80 例体会。按摩与导引　2002; 18(2): 13
4　郑良义。卧位旋转复位法治疗根型颈椎病。中医骨伤　1999; 12(4): 61
5　白光。扳颈手法治疗颈推病引起的并发症原因探讨。包头医学　1998; 22(3): 110
6　梅继文，梁文杰，韦贵康。颈椎病手法意外 21 例临床分析。中国中医骨伤科杂志　2003; 11(5): 53
7　赵振彰。生理性关节弹响的实质和临床意义。按摩与导引　1997; (6): 4
8　李义凯，赵卫东，钟世镇。两种颈部旋转手法"咔嗒"声的比较研究。中医正骨　1998; 10(6): 9
9　Triano J. Studies on the biomechanical effect of a spinal adjustment. J Manipulative Physiol Ther　1992; 15: 71
10　姜宏。牵引推拿对颈椎生物力学影响的实脸研究。中医博士论文集粹。上海：上海中医药大学出版社，1996; 170
11　孙树椿，易金根，张军。旋转手法治疗颈椎病的临床和实验研究。中国针灸　1995; (2): 58
12　Triano J. Studies on the biomechanical effect of a spinal adjustment. J Manipulative Physiol Ther　1992; 15: 71
13　李义凯，钟世镇。颈椎管侧弯实验形态学变化及其临床意义。解剖与临床　1997; 2(3): 98
14　KwachukGN, Herzog W. Biomechannical characterization(fingerprinting) of five novel methods of cervical spine manipulation. J Maninnlative PhvAinl Ther　1993; 16: 573
15　李义凯，谢秋屏，钟世镇。欧美脊柱推拿基础研究进展。按摩与导引　1998; 14(4): 1
16　姜宏，施杞，王以进。颈椎失稳临界值和极限强度的测量。医用生物学　1997; 12(4): 224
17　姜宏，施杞，王以进。旋转手法对颈椎间盘黏弹性影响的实验研究。中国中医骨伤科杂志　1999; 7(1): 4
18　姜宏，颈椎急慢性扭伤的生物力学研究概况。中医正骨　1996; 8(6): 31
19　陈述之，陈湘昂。颈椎病的手法治疗及其安全性问题。中国康复　1992; 10(3): 134
20　梅继文，梁文杰，韦贵康。颈椎病手法意外 21 例临床分析。中国中医骨伤科杂志　2003; 11(5): 53
21　李义凯，张云昆，钟世镇。颈部旋转手法对椎动脉流速影响的研究。中国中医骨伤科杂志　1997; 5(5): 7

?1994-2014 China Academic Journal Electronic Publishing House. All rights reserved.　http://www.cnki.net
