# Supplementary material for: Kinematics Analysis of Cervical Rotation-Traction Manipulation Measured by a Motion Capture System
Source: Evid Based Complement Alternat Med. 2017 Oct 11;2017:5293916. doi: 10.1155/2017/5293916 (PMC5660796; doi:10.1155/2017/5293916)
Supplement: Supplementary file 1 — Procedure of CRTM, the related result of CRTM, the related reference. [file 5293916.f1.zip › Supplementary Material/Appendix 3.pdf]

。名医方技。

孙树椿主任医师治疗颈椎病的手法经验

张　军
(中国中医研究院骨伤科研究所　北京　100700)

关键词　颈椎病　名医　治疗经验
中图分类号: R681.5　　文献标识码: B　　文章编号: 1005-0205(2000)04-0060-03

孙树椿主任医师是国内著名骨伤科专家,现任中国中医研究院首席研究员、博士生导师、主任医师,中国中医研究院骨伤科研究所颈椎病研究室主任,中国中医研究院望京医院脊柱二科主任,国务院政府特殊津贴获得者,曾任中国中医研究院骨伤科研究所所长。早年大学毕业后,从师于当代骨科名老中医刘寿山老先生门下,尽得刘老理伤正骨手法真传。近几十年来,一直从事骨伤科的临床、科研和教学工作,对骨伤疾病有深刻的认识,曾多次为国内外知名人士及政府要员诊治疑难病症,常有手到病除的疗效。善于灵活运用中西医两种方法,进行诊断和治疗疾病。同时,注意突出中医特色,努力挖掘和发扬祖国传统医学特色。积极提倡中医骨伤科手法的治疗,注意筋伤手法的研究和整理。近年来,对颈椎病进行了较深入的研究和探讨,提出了一整套对颈椎病的诊断和治疗独特的方法。同时,将自己多年的临床经验和心得体会,毫不保留地进行传授。指导的学生万余人,培养中医骨伤手法治疗颈椎病博士研究生 2名,硕士研究生 8名。作者自 1994年师从孙树椿老师,亲聆教悔,受益匪浅,深深体会到中医骨伤科学的博大精深,现将部分的心得体会汇报如下。

1　手法的操作要领

老师的手法主要源于著名老中医刘寿山老先生,老师在继承刘老先生的经验同时,运用现代的解剖生理学和病理生理学知识对其进行了规范整理。在保持疗效的基础上,简化精练了手法,使之便于学习掌握和推广应用。强调运用中医骨伤手法治疗颈椎病,是祖国医学的一大优势,手法作用于局部体表可以影响到所连属的脏腑、组织,调节

与衣原体感染有关[2]。外斐氏试验是否可作为 RS征常规检查指标,有待传染病学家及免疫学家关注。

RS临床除有眼—— 尿道—— 关节炎典型三联征外,尚可有龟头炎,溢脓性皮肤角化病、口腔损害及全身表现。不典型的 RS要与类风湿性关节炎、强直性脊柱炎、银屑病性关节炎、痛风、淋病性及其它感染性关节炎、急性风湿热、血清病的关节症状,系统性红斑狼疮等相鉴别。

本病例早期到多家医院诊治均误诊,其原因分析如下: (1)对该病缺乏认识,该病多见于青壮年男性,儿童少见,且临床典型三联征出现的时间不一致。尿道炎、结膜炎易被忽视。或将结膜炎、尿道炎视为发热的反应。(2)患者往往因为此疾病的不同表现形式而到各科诊治,专科医生往往对初发感染有所忽视,郁于专科知识而欠缺全面思考。因于骨科历年收治的关节炎均以风湿多见。因而凡遇关节红肿热痛、功能障碍者都把风湿列为首位。

我们认为本病诊断应注意以下几点: (1)凡是菌痢后罹思关节炎者,应高度警惕此病。(2)对长期发热,关节肿痛、血 WBC增高, ESR增快,而 Rf阴性, ASO(500u时应仔细录间病史及做详细的体检。(3)当病史或体征证实存在三联征,又除外沙门氏、痢疾菌属感染后关节炎者即可确诊。(4)临床各有关学科密切协作以期减少误诊、漏诊。

参考文献

[1] 施桂英.赖特综合征.人民军医, 1994, 421(12): 68~69
[2] 章俊爵,孙泰安,蒋晓天.儿童 Reiter综合征 5例误诊分析.南通医学院学报, 1995, 15(4): 617~634

收稿日期: 2000-04-20
作者简介: 张军(1967-),男(汉族),吉林省长春市人,医学博士,主治医师。

?1994-2015 China Academic Journal Electronic Publishing House. All rights reserved.　http://www.cnki.net

机体的生理功能,使百脉疏通,五脏安和,达到治疗效果。正如《医宗金鉴。正骨心法要旨》记载:“因跌扑闪失,以致骨缝升错,气血郁滞,为肿为痛,宜用按摩法。按其经络,以通郁闭之气,以散瘀结之肿,其患可愈”。老师治疗颈椎病的手法大致包括以下三部分:即预备手法、治疗手法及善后手法。

1.1 预备手法

此手法包括揉捻法和滚法,其目的在于松解痉挛僵硬的颈肩肌群,促进局部血液循环,使之收到舒筋通络,宣通气血,解痉镇痛的效果,同时也为下一步手法的运用打好基础。

1.1.1 揉捻法 操作时,患者正坐,术者位于患者身后,用大鱼际、掌根或指面交替在两侧颈部(肌肉处)或某一穴位上,自上而下做回旋揉捻,以患者感觉轻微的酸痛,可以忍受为度,做轻柔和缓的环旋运动。其作用力可达皮下组织,也可深达肌层。具有解痉镇痛,松解软组织粘连的作用。指或掌应紧贴皮肤不移、使皮下组织随指或掌的揉动而滑动,不要在皮肤上来回搓动,用力要均匀,速度不宜过快,频率每分钟约 50~100次,在压痛点可做重点揉捻,时间应稍长一些,一般每侧施同样手法。临床上可分为指揉法和掌揉法二种:指揉法:以手指腹侧面按于某一部位或穴位上,作小幅度的环旋揉动。指揉法作用面小,力量较深在稳重。掌揉法以掌根部或大鱼际为着力点,腕部放松,以腕关节连同前臂作回旋运动,着力面大,刺激要和缓舒适。

1.1.2 滚法 操作时,患者正坐,术者位于患者身后,手呈半握拳状,以 2~5指的近端指间关节为支点放于患处,以腕关节带动,作均匀的来回摆动,来完成滚动的动作。着力点要深,用力要均匀柔和,力量要推进肌肉深部。是用手背部在体表一定部位作连续往返滚动的一种手法,具有促进血液循环,舒筋活络,解痉止痛,消除肌肉疲劳的作用。此手法用于头颈根部及双肩部尤为适宜。操作时,要以腕的灵活摆动带动掌指关节部的运动,滚动时腕关节要放松,滚动速度一般以每分钟 60~100次为宜,并要有轻重均匀交替、持续不断的压力作用于治疗部位上,着力点必须紧贴皮肤。切忌来回摩擦而造成皮肤损伤。

1.2 治疗手法

患者取正坐位,术者立于患者身后,稍微侧身。下以右旋为例:用右手或右前臂置于患者颌下,左手托住枕部,轻提并且做颈部旋转运动 2~3次。目的在于使患者颈部肌肉放松,然后上提,牵引颈部,并使其保持中立位,牵引的同时将患者的头颈右旋至有固定感时,右手或右前臂快速发力旋转颈部,此时即可听到一连串的弹响声,一般响声清脆者疗效为佳。之后以同样手法向左侧旋复一次,手法完毕。此手法的要点在于手法的全过程都是在轻度牵引下进行。在应用本手法时,要稳、准、轻柔,不可粗暴。旋转要适度,力量不宜过大。本手法是治疗颈椎病的重点手法。其目的在于分解颈椎小关节的粘连,纠正颈椎关节的错缝,减轻关节负压,并且可以加宽狭窄的椎间隙,扩大狭窄的椎间孔,使颈椎恢复正常的生理曲度,从而缓解由于颈椎病变对神经根、血管及周围软组织的压迫和刺激而引起的症状。

1.3 善后手法

包括劈法、散法、拿法及归合法等。其目的为放松颈肩部肌群,进一步解除肌肉痉挛,改善血运,增加局部血液循环,消除软组织的炎性反应。从而起到流风通络、消炎止痛、调和气血之功。

1.3.1 劈法 患者取正坐位,术者立于患者身后。双手五指分开放松,以手掌尺侧,劈打双肩部及背部 1分钟。

1.3.2 散法 用双手掌指桡侧在两侧颈部(肌肉处)交错散之,用力按压之后,散法效果才好。再从上至下到肩部时,改用掌侧散之,对两侧肩背部肌肉也要散到,做 2~3遍。

1.3.3 拿法 用拇指和掌与其余四指的指腹相对用力,在肩部拿捏,拇指做环行运动。此法行 1~2分钟。

1.3.4 归合法 双手交叉,以两手掌大小鱼际至患者颈部及肩部相对归济,自上而下,做 2~3遍。根据患者的不同情况,可在上述手法基础上,加用扣法。抖肩法及捋顺法等,以颈肩部自觉发热为好,从而完成整个手法。

我们曾对老师诊治的 286例颈椎病人(神经根型 87例,椎动脉型 64例,脊髓型 19例,交感神经型 11例,颈型 49例,混合型 56例)进行了观察总结,其中临床治愈 163例,临床显效 72例,临床好转 38例,无效 12例。治疗次数最多 30次,最少 1次,平均为 9.1次。其中治愈、显效者共 235例(82.2%),临床总有效率为 95.8%。

2 经验体会

2.1 “法之所施,使患者不知其苦,方为手法”。

老师在手法运用中充分强调持久、有力、均匀、柔和。但在临床中如何才能做到使患者不知其

?1994-2015 China Academic Journal Electronic Publishing House. All rights reserved. http://www.cnki.net

苦病变消失呢?那就是要很好地作到轻重结合、刚柔相继,先轻后重,逐渐增加力度,刚寓于缓稳操作之柔中,刚柔相济,力要缓稳柔和,以调节机体的生理、病理状态,从而达到治疗和缓解病患。“持久”指手法持作时能持续运用一定时间,保持动作和力量的连贯性,同时使该部位产生感应,对某些需重点治疗的穴位和部位,更需维持较长时间的操作。“有力”是指手法具有一定的力度,包括固定部位的压力和运用的功力,同时根据治疗的对象、病症的虚实、施治的部位来决定,使手法轻而不浮,重而不滞。“均匀”是指手法动作的节奏性和用力的稳妥性,动作频率要有节奏而协调,用力要稳。“柔和”是指手法动作的节律协调及用力的均匀缓和,是手法技巧和力量的完美结合。老师指出手法的治疗是纠正颈椎失稳,消除致痛因素,而不是去破坏颈椎的平衡系统,严禁浮躁粗暴。正如《医宗金鉴·正骨心法要旨》所说:“法之所施,使患者不知其苦,方为手法”。

2.1.2 “手随心转,法从手出”

老师指出手法要经过长期的磨炼才会具有一定的“功力”,达到“深透”的效果。这时就会发现病人深部的病变结节或条索,多表现发凉的感觉,医者之手如烧红的铁棒,会使病变深部的结节或条索慢漫化掉,最终达到好转治愈。说实在的,手法的功力能达到这个境界是不易的。除了日常的苦练外,还应仔细体会手法的技巧,非一时一日之功,需要长久的专研才可能达到。正如《医宗金鉴·正骨心法要旨》所说的:“一旦临近,机触于外,巧生于内,手随心转,法从手出。”

2.1.3 “气血不和、百病乃变化而生”

老师在颈椎病的治疗中十分强调气血的关系,指出气有所病必及于血,血有所凝必影响气。气血相辅相成,互相依附,循行全身,周流不息,是人体生命活动的物质基础。若平衡失调,气滞血瘀于脉络,则成“离经之血”和“污秽之血”,可导致“不通则痛”。因为瘀积不散,为肿为痛,血不活者瘀不去,瘀不去则痛不除。中医骨伤手法通过逐瘀血,通经络,达到和血止痛,符合中医学的“经络、气血不通则痛”,“治痛必治瘀”的理论。通过准备手法解除颈部肌肉的痉挛,疏通气血,舒筋活络,消除充血、水肿,改善局部气血运行,疏通活络,消除离经之血;旋转复位手法可使“骨对缝”、“筋入槽”,减轻关节内的负压,解除因患椎失稳对血管、神经和脊髓产生的刺激或压迫;善后手法,可以顺筋通络,放松肌肉,通利关节。通过手法的治疗改善颈部的气血运行情况,使气血调达,从而对颈椎病起到系统的调治。

2.1.4 手法的注意事项及禁忌

老师在临床诊断治疗时特别强调:(1)由于个体的生长发育、生活环境和病理生理条件的不同,对手法的反应不完全相同。因而手法的力度应因人而异,必须与治疗对象、病症虚实以及治疗部位联系起来。因为人有男女老少之别,症有虚实久暂之分,治疗部位有大小深浅之不同。因此何种手法操作时间的长短,手法力量的轻重以及掌握治疗的重点等,都要因人、因病、因部位的不同再灵活运用。(2)对手法的操作步骤应心中有数,要注意局部的解剖结构和关节的正常活动范围,用力要轻重适当,避免因过猛过重引起神经血管或关节结构的损伤,而加重病情。(3)对于损伤较重或急性发作期者手法要轻柔;对于慢性劳损者手法可重一些。在手法应用过程中要注意观察患者的表情,询问其自我感觉,随时调整手法强度。手法操作要做到熟练灵活,敏捷准确,尽量使患者不受痛苦或少受痛苦。(4)应用手法时,思想要集中,态度应从容沉着,取得患者的信赖和配合,减轻患者的紧张情绪。同时患者的体位也要适当,颈部肌肉应充分放松。(5)应明确诊断,对病情应有充分了解,如病位、损伤程度、病程长短、病情轻重、有无神经血管损伤和骨折等。中医的手摸心会还应与影像学检查相结合。如颈部肿瘤、颈椎骨折和骨结核患者,禁用手法;老年性骨质疏松、颈椎退变骨桥形成或椎间孔狭窄明显,椎动脉扭曲严重,严重的脊髓型颈椎病或伴有严重的冠心病者,手法操作宜轻柔,不宜过重。

此外,在颈椎病治疗的同时,老师指出枕头的合适情况,也是长期困绕颈椎病患者的一个重要因素。白天人体的脊椎处于紧张状态,肌肉容易疲劳。夜晚休息时,如果枕头的高度不合适,过高或过低,均会牵扯颈部的肌肉,使之一夜处于紧张疲劳状态,会引起病情的反复或加重。所以,枕头的适合高度是指人体无论在仰卧还是在侧卧时,要保证颈椎与脊柱保持水平,这样才可以使颈部肌肉放松,从而更好地保证治疗效果,减少复发。

?1994-2015 China Academic Journal Electronic Publishing House. All rights reserved. http://www.cnki.net
