# Supplementary material for: Kinematics Analysis of Cervical Rotation-Traction Manipulation Measured by a Motion Capture System
Source: Evid Based Complement Alternat Med. 2017 Oct 11;2017:5293916. doi: 10.1155/2017/5293916 (PMC5660796; doi:10.1155/2017/5293916)
Supplement: Supplementary file 1 — Procedure of CRTM, the related result of CRTM, the related reference. [file 5293916.f1.zip › Supplementary Material/Appendix 5.pdf]

·临床论著·

两种颈部旋转手法“咔嗒”声的比较研究[1]

第一军医大学临床解剖学和医用生物力学研究所(广州 510515)
李义凯[2] 赵卫东 钟世镇

提要 为提高颈部旋转手法的准确性,应用定向式微型麦克风记录两组患者在施颈部旋转手法作用时发出的“咔嗒”声。结果表明,颈部旋转手法主要作用在旋转侧,只有少部分是作用在旋转的对侧。定点旋转复位法作用范围小,作用点相对集中;端提旋转复位法作用的节段较多,作用点相对分散。说明旋转手法主要作用在旋转侧,并非旋转的对侧;端提旋转复位法作用范围较广,对上颈段病变的疗效较好;而定点旋转复位法作用范围较为局限,适用于下颈段病变;旋转时“咔嗒”声的出现,预示着关节运动达到了极限。

主题词 颈椎病/治疗 推拿 旋转复位手法 手法/方法 对比研究

颈部推拿手法种类繁多,但大数离不开颈部的旋转手法。临床上常用以及教科书介绍较多的颈部旋转手法是端提旋转手法和定点旋转手法两种。后者是从冯天有的定点旋转复位手法衍化而来的,基本内容雷同。旋转手法在治疗一些颈部疾病时,常有手到病除之效。但实践中也发现一些医者在使用颈部旋转手法时,并没有遵照辨证施治的原则,而是不论上颈段还是下颈段的病变,均采用固定的旋转手法。在使用旋转手法时,常可闻及患者的颈椎发出“咔嗒”声。对此,手法治疗者认为这是旋转手法成功的标志,而患者也以此声响来衡量术者水平的高低,但对旋转过程中产生的“咔嗒”声在临床上的意义,则有不同的看法[1]。一般认为“咔嗒”声响与术者所使用的手法、力量以及患者本身的解剖结构等因素均有一定的关系。对上述两种常用的颈部旋转手法操作时产生的“咔嗒”声与头颈部旋转方向等之间的关系,还未见研究报道。为提高颈部旋转手法的准确性,我们对上述两种颈部旋转手法进行了比较。现总结报告并分析讨论如下。

1 材料和方法

1.1 临床资料 本组 60例均为门诊患者。年龄 26~47岁,平均 35岁。男女之比为 23∶37 其中颈椎病 26例(主要为神经根型颈椎病),落枕 18例,颈枕部疼痛不适 16例。所有患者在行手法前均拍摄了颈部正侧位或多斜位 X线片并进行了脑血流图等检查,以排除严重的椎-基底动脉供血不足、肿瘤、骨质疏松和其它一些手法禁忌症。随机将患者分为两组,组 1为端提旋转复位法,组 2为定点旋转复位法。每组 30例患者。为减少手法操作中的误差,所有旋转手法均由一人操作完成。组 1中,颈痛伴有上肢麻木者 14例,单纯性颈痛 10例,颈部不适者 6例;男女之比为 11∶19;其中伴有眩晕、耳鸣等症状者 13例。组 2中,颈痛伴有上肢麻木者 12例,单纯性颈痛者 1例,颈部不适者 7例;男女之比为 12∶18;其中伴有眩晕、耳鸣等症状者 16例。

1.2 采集方法 将两个定向式微型麦克风分别置于患者颈后部的皮肤上(平胸锁乳突肌后缘中点水平),相当于 $C_{4\sim5}$小关节的正后方[2]。用胶带将麦克风固定,防止皮肤与麦克风之间发生相对运动。然后将麦克风与立体录音机的左右通道相连,根据录音机的左右通道确定麦克风的左右侧并做标记。采集时按照患者的序列,用录音磁带记录每次旋转手法时,患者颈椎发出的“咔嗒”声响。

1.3 手法操作 (1)端提旋转复位法:患者坐位,主动放松颈项部肌肉。术者一手抵住患者头侧后部,另一手抵住对侧下颌部。做几次轻缓的颈部摇晃之后,在颈部微向前屈拔伸时,两手同时用力作相反方向的扳法迅速向患侧加大旋转幅度,手法要稳而快速,旋转幅度要在患者能忍受的限度内。(2)定点旋转复位法:患者端坐,颈部自然放松,双手触诊检查偏歪的棘突,然后向棘突偏歪侧主动旋转至最大限度。术者一手拇指顶推偏歪的棘突,其余四指扶持颈部,另手前臂掌面紧贴下颌体。掌心抱住后枕部。将抱头的手向上牵拉并向棘突偏歪侧旋转头颈部,与此同时推顶棘突的拇指突然发力。此时多可听到一声“咔嗒”声,觉得拇指下的棘突轻度位移。

1.4 临床疗效 推拿后立即询问患者的自我感觉,症状是加重还是减轻,由此判断关节声响与疗效之间的可能关系。

1.5 录音分析 根据左右侧麦克风所记录的“咔嗒”声响,分析旋转与“咔嗒”声响的关系。

2 结果与分析

两组患者在行旋转手法时,除 1例外,所有患者在旋转颈部时均产生了“咔嗒”声响。

2.1 端提旋转复位法 采用本法的 30例患者均产生“咔嗒”声响。共发出 63下“咔嗒”声响,其中 9例为单下声响,13例为两下声响(其中 3例有一响声是出现在对侧),4例出现三下声响(其中 1例有两声响出现在对侧),4例出现四下声响。30例中 26例的声响是出现在头颈的旋转侧,4例是双侧均有,其对侧声响是在旋转侧发出声响后随后出现的。在轻微转动患者头颈时,有 3例其头旋转侧发出“咔嗒”声,但在旋转时又发出声响。

---

1 本课题为军队医药卫生科研基金资助项目
2 现工作单位为第一军医大学中医系

?1994-2016 China Academic Journal Electronic Publishing House. All rights reserved. http://www.cnki.net

2.2 定点旋转复位法 采用本法的 30例患者,除 1例外,均产生"咔嗒"声响。29例患者共发出 45下声响,其中 16例为单个声响,10例为两下声响(其中 1例有一响声是出现在对侧),3例 3下声响。29例中 28例的"咔嗒"声响是出现在头颈的旋转侧,1例为双侧均有,但对侧声响是随后出现的。在旋转头颈至极限时有 6例患者出现"咔嗒"声响,但在快速旋转其头颈时又发出声响(表 1)。

表 1 两种颈部旋转手法咔嗒声的比较(例数)

组别	单个声响	2下声响	> 2下声响	旋转侧	旋转对侧	双侧
组 1	9	13	8	26	4	4
组 2	16	10	3	28	1	1

表 2 关节声响与临床疗效之间的关系(例数)

组别	颈痛		颈部不适		上肢麻木		眩晕耳鸣	
	疗前	疗后	疗前	疗后	疗前	疗后	疗前	疗后
组 1	10	4	6	1	14	11	13	4
组 2	11	5	7	3	12	6	16	8

两组患者在颈部推拿过程中出现关节咔嗒声响者,绝大多数表示颈部在旋转后有轻松感(见表 2)。定点旋转复位法对下颈段病变所致的神经根型颈椎病的疗效较好,特别是对上肢麻木。而端提旋转复位法对与上颈段病变密切相关的眩晕、耳鸣等症状有较好的缓解作用。

3 讨论及结论

3.1 旋转手法的作用机制 推拿界有一些特定的专业术语,如:脊椎关节半脱位、小关节综合征和小关节紊乱等一些专业名词。虽然每一种术语均有其特定含义和定义,但其基本的内容均包括椎体关节运动受限或异常。对其治疗,手法推拿治疗者认为,推拿特别是旋转手法可恢复病变关节的正常运动。对颈部病变的患者,最常选用的治疗手法是旋转复位手法。旋转手法在临床上使用广泛,一般认为旋转手法的作用机制有四种:① 解除嵌顿或嵌压的滑膜皱襞;② 突然的旋转牵拉使处于高张力的肌肉得到缓解;③ 松解关节或关节周围的粘连;④ 纠正关节错位,恢复关节的正常运动[3]。

3.2 "咔嗒"声的产生机制 在做关节推拿时,常可听见由关节发出的"咔嗒"一声清脆音。对此医生和患者都较为重视,因为在治疗时若听到关节发出"咔嗒"声响,医生即认为是合臼,少数文献对"咔嗒"声响的发生机理有不同的推测[4]。有人认为是两个关节面软骨之间的轻快磨擦所致;也有人认为是嵌顿的滑膜复位造成的[5 6]。但多数认为是在快速的旋转过程中,关节内气体的迅速流动所致,这部分研究主要集中在掌指关节的"咔嗒"声响上,认为掌指关节的"咔嗒"声响与脊柱产生"咔嗒"声响没有多大的区别[7]。近来的一些研究结果支持这种理论,但具体的机制有待进一步研究。从力学观点来分析,做脊柱推拿手法时要使脊柱关节产生"咔嗒"声,必须具备旋转力,即杠杆力。推拿时,脊柱关节出现"咔嗒"声,可以说明推拿的旋转力已经作用到脊柱关节,使其产生了活动;并且使被旋转的关节处于关节运动的极限状态。

3.3 "咔嗒"声的临床意义 表 2显示,推拿时"咔嗒"声的出现与临床疗效有一定的关联,应用手法治疗后使颈椎小关节出现"咔嗒"声后,一些临床症状会立即得到缓解。有人认为此声响非常重要,将推拿过程中产生"咔嗒"声响视为手法治疗成功的关键。患者也认为有此声响者疗效好,无此声响者疗效差。但人认为这仅是心理作用而已。虽然推拿医师比较重视推拿时发出的声响,但临床上推拿治疗者很少考虑颈部旋转的方向与颈部推拿时发出"咔嗒"声的关系。根据本研究结果,颈部旋转手法主要作用在旋转侧的小关节上,只有少部分是作用在旋转的对侧。定点旋转法作用范围小,作用点相对集中;端提旋转复位法作用的节段较多,作用点相对分散。从关节声响与临床疗效之间关系可看出,对下颈段病变,如神经根型颈椎病的治疗上,定点旋转复位法优于端提旋转复位法。因而在临床上,要根据不同的临床特点,选择不同的手法,使颈部旋转手法的应用有一定的针对性。

要使脊柱推拿时出现关节的"咔嗒"声,就必须有正确的操作手法。除一定力量外,在旋转时还要注意到旋转的角度和旋转的作用点。若旋转时出现较多的关节"咔嗒"声响,这只是表明旋转作用力的范围较大,旋转手法的操作带有一定的盲目性。出现关节"咔嗒"声表明该关节运动已达到极限,此时如果加大旋转力量可能会造成伤害。

3.4 结论 旋转手法的主要作用点是在旋转侧,并非旋转的对侧。提端旋转复位法作用的范围较广,对上颈段病变的疗效较好。而定点旋转复位法作用范围较为局限,适用于下颈段病变。旋转时"咔嗒"声的出现预示着关节运动达到了极限。

4 参考文献

1 Herzog W, Zhang YT, Conway PJ, et al. Cavitation sounds during manipulative treatments. J Manipulative Physiol Ther 1993; 16 523

2 John W, Reggars, Henry P, et al. Analysis of zygapophyseal joint cracking during chiropractic manipulation. J Manipulative Physiol Ther 1995; 18(2): 65

3 Paul G, Shekelle. Spine update spinal manipulation. Spine 1994; 19(7): 858

4 Meal GM, Scott RA. Analysis of the joint crack by simultaneous recording of sound and tension. J Manipulative Physiol Ther 1986; 9 189

5 DiGiovanni EL. In DiGiovanni EL, Schlowitz S, eds. An steopathic approach to diagnosis and treatment. Philadelphia: J B Lippincott 1991; 6

6 Sandoz R, Some physical mechanisms and effects of spinal adjustments. Ann Swiss Chiropractors Assoc 1976; 6 90

7 Woods GW, West VC. A comparision of temporomandibular joint sounds with the sounds from. ther joijts of the body. J Craniomandibular Prac 1986; 4 345

(1997-0-0收稿 1998-0-0修回)

?1994-2016 China Academic Journal Electronic Publishing House. All rights reserved. http://www.cnki.net

THE JOURNAL OF TRADITIONAL CHINESE ORTHOPEDICS AND TRAUMATOLOGY

Volume 10 November 1998 Number 6

ABSTRACTS OF ORIGINAL ARTICLES

THE TREATMENT OF RAT SPINAL CORD INJURY BY MODIFIED " BUYANG HUANWU TANG": AN EXPERIMENTAL STUDY

Yang Yundong 杨运东, Feng Qiuzhen 冯秋珍, Wei Yuling 魏玉玲, et al

Hubei College of Traditional Chinese Medicine, Wuhan 430061

Animal models of complete injury of T_{10} spinal cord were made by Allen's equipment and a force of 10 x 10 g. cm in 20 healthy S-D rats. The 20 animals were randomly divided into two groups: TCD group and control group, 10 for each. The ultrastructure changes, spinal nerve function, and histological and morphometric analyses were taken as the observed indices, and observed through experiments. The results showed that TCD group had an advantage over the control in the ultrastructural recovery of neuron cells and myelin sheaths and the analysed results of the spinal-nerve function and histomorphometry. Through analyses, it was believed that modified " BUYANG HUANWU TANG" (Decoction Invigorating Yang for Recuperation) functions to prevent the secondary injury of spinal cord and stimulate the repair and regeneration of neurons and injured nervous fibres.

Key Words: spinal cord injury/TCM therapy modified " BUYANG HUANWU TANG" /therapeutic application spinal cord injury/untrastructure experimental study animal rat

(Original article on page 3)

AN EXPERIMENTAL STUDY ON ANTI-INFLAMMATORY AND ANALGETIC EFFECT OF " JINGFU TIE"

Ba Yan 巴 艳, Li Genlin 李根林, Zheng Fuzeng 郑福增, et al

Henan Medical University, Zhengzhou 450052

Through experimental study, it was suggested that " JINGFU TIE" (JFT) (Plaster for Cervical Spondylosis) has a significant inhibitory effect on mouse ear edema and rat foot edema and granuloma ($P < 0.01$), and functions to improve pain threshold. JFT effect is stronger and starts quicker than that of Plaster for Strenthening Bone and Joint, and is enhanced with increased dosage. This study has fully proved the anti-inflammatory and analgetic effect of JFT and offered a reliable theoretic basis for its clinical application.

Key Words: " JINGFU TIE" /pharmacology anti-inflammation/drug action analgia/drug action experimental study animal rat

(Original article on page 5)

ANTI-IRRITABLE EFFECT OF " JIAWEI YIQI WAN" ON MICE AN EXPERIMENTAL STUDY

Wang Sujing 王苏静, Guo Yanxing 郭艳幸, Chang Shiqing 常世卿, et al

Luoyang Orthopedic-Traumatological Hospital, Henan Province, Luoyang 471002

" JIAWEI YIQI WAN" (JYW) (Modified Pills for Supplementing Qi) is one of the internal-therapy drugs in Pingle Orthopedics and Traumatology. In order to observe the action mechanism of its anti-irritable effect, swimming, anoxia-tolerant, thermotolerant and cryotolerant experiments were carried out on mice. The results showed that JYW functions to lengthen the swimming time of the mice, and has a significant effect on anoxia-tolerance, thermotolerance, and cryotolerance. So, JYW was proved to improve the ability of the organism to resist irritability.

Key Words: " JIAWEI YIQI WAN" /pharmacodynamics anti-irritable effect experimental study animal rat

(Original article on page 7)

A COMPARATIVE STUDY ON THE CRACKINGS DURING TWO ROTATORY MANIPULATIONS OF THE NECK

Li Yikai 李义凯, Zhao Weidong 赵卫东, and Zhong Shizhen 钟世镇

Institute of Clinical Anatomy and Medical Biomechanics, First Military Medical University, Guangzhou 510515

?1994-2016 China Academic Journal Electronic Publishing House. All rights reserved. http://www.cnki.net

In order to improve the accuracy of the rotatory manipulations of the neck, the crackings during two of the manipulations were recorded by a microphone positioned at two sides of the neck and compared in patients of two groups. The results showed that neck rotatory manipulations act mainly on the rotatory-side small joints but on the opposite to the rotatory side only in a few cases. Fixed-point lifting-supporting rotation (FLSR) has a small action range and centralized action points, while lifting-supporting rotation (LSR) has many action segments and discentralized action points. It was concluded that the rotatory manipulations act mainly on the rotatory side, not on the opposite, and that LSR has a wider action range and a better therapeutic effect on cervical upper-segment lesions, while FLSR has a limited action range and is suitable for cervical lower-segment lesions. The appearance of the crackings during rotation predicts the limit of joint motion.
Key Words: cervical spondylopathy/treatment massage rotatory manipulation/methodology comparative study
(Original article on page 9)

A EXPLORATION ON THE RELATIONSHIP BETWEEN THE POSTERIOR RAMUS OF LUMBAR SPINAL NERVES AND LUMBAGO AND SCELALGIA

Xiao Luwei 肖鲁伟, Tong Peijian 童培建, and Zhao Wanjun 赵万军
Zhejiang College of Traditional Chinese Medicine, Hangzhou 310009
A closed relationship between the posterior ramus of lumbar spinal nerves and lumbago and scelalgia was revealed through autopsy and local-blockade treatment and observation of lumbago and scelalgia. Since the posteromedial and posterolateral rami of lumbar spinal nerves, in their courses, pass through osseofibrous foramina and canal or lumbodorsal-fascia fissure, respectively, and the former has an "S"-shaped course in osseofibrous canal in particular, the long-term repeated drawing, sprain and bruise of the tendon and fascia forming the above foramina and canal may result in clinical onset of lumbago and scelalgia.
Key Words: posterior ramus of lumbar spinal nerve/anatomy lumbago and scelalgia/pathology
(Original article on page 11)

THE EFFECT OF SPINAL TRACTION ON THE REHABILITATION OF ANKYLOSING SPONDYLITIS

Li Xinzhong 李新忠, Yang Xue 杨　雪, Cao Zaijie 曹在杰, et al
Linyi Hot-Spring Sanatorium for Chinese Miners, Shandong Province 270032
In order to study the effect of spinal traction on the rehabilitation of ankylosing spondylitis, 134 cases of the disease were randomly divided into the observed group and the control group. The former ($n = 74$) were treated by pharmacotherpy and physotherapy and, at the same time, by spinal traction; the latter ($n = 60$) were treated only by pharmacotherapy and physotherapy. The results showed that compared with the control, the observed group obtained better therapeutic effects with a higher excellent-good rate ($P < 0.05$) and shorter treatment course ($P < 0.01$). It was believed that spinal traction has an active effect for improving therapeutic effect, shortening treatment course and preventing deformity.
Key Words: spondylitis, ankylosing/rehabilitation and treatment spinal traction comparative study
(Original article on page 13)
(王芳轩译校)

本卷终。感谢大家以往对我们的关心、爱护和大力支持！希望今后给予更多的关注……。

THE JOURNAL OF TRADITIONAL CHINESE ORTHOPEDICS AND TRAUMATOLOGY

Bimonthly
Established 1989
Chief Editor
Guo Weihuai
Managing Editor
Qin Kefeng

Supervised by: The State Administration of Traditional Chinese Medicine, THE PEOPLE'S REPUBLIC OF CHINA
Sponsored by: Luoyang Institute of Traditional Chinese Orthopedics and Traumatology of Henan Province
Edited by: The Editorial Board of《The Journal of Traditional Chinese Orthopedics and Traumatology》
Published by: The Editorial Office of《The Journal of Traditional Chinese Orthopedics and Traumatology》. Add. No. 1 South Qiming Road, Luoyang, China. Post Code: 471002
Distribution Range: public
Domestic Subscription: Luoyang Post Office
Foreign Subscription: China International Book Corporation (P. O. Box 399, Beijing Code: BM 4182), THE PEOPLE'S REPUBLIC OF CHINA
Address for Subscription: Local Post Offices in China. Code: 36-129
Printed by: Yanshi Printing House, Henan Province, China

Volume 10 **Number** 1 **Publication Date**: **November** 20, 1998 **Code**: 36-129 (**BM** 4182)
Subscription Price (for single copies): **Domestic** 4.80 **yuan RMB**; **Foreign** 10.00 **USD**

?1994-2016 China Academic Journal Electronic Publishing House. All rights reserved. http://www.cnki.net
