# Supplementary material for: Kinematics Analysis of Cervical Rotation-Traction Manipulation Measured by a Motion Capture System
Source: Evid Based Complement Alternat Med. 2017 Oct 11;2017:5293916. doi: 10.1155/2017/5293916 (PMC5660796; doi:10.1155/2017/5293916)
Supplement: Supplementary file 1 — Procedure of CRTM, the related result of CRTM, the related reference. [file 5293916.f1.zip › Supplementary Material/Appendix 6.pdf]

·临床研究·

颈部旋转相关极限体位对椎动脉血流的影响

张法尧[1,2△] 赵树森[1] 朱立国[2]

［摘要］ 目的:使用多普勒超声评价颈部旋转相关极限体位对椎动脉血流的影响。方法:对 40 个健康成人年龄在 19～21 岁(平均 20.2 岁),分别在颈部中立位、同侧旋转极限,同侧屈曲旋转极限,同侧后伸旋转极限,对侧旋转极限,对侧屈曲旋转极限,对侧后伸旋转极限,卧位,卧位同侧旋转极限,卧位对侧旋转极限等体位下进行椎动脉的流速,管径多普勒超声测量。测量椎动脉位于颈 3～4 节段,血流量和总血流量通过计算获得,总血流量为同一体位下双侧椎动脉流量和。对包括椎动脉流速,管径,流量及颈部活动角度等数据进行统计分析。结果:后伸旋转极限体位的总血流量大于立位旋转极限位,屈曲旋转极限位,卧位旋转极限位,差异有统计学意义($P<0.05$),而后三者之间差异无统计学意义($P>0.05$);与中立位比较,旋转极限位和屈曲旋转极限位时,对侧椎动脉血流量均减少,差异有统计学意义($P<0.05$),后二者差异无统计学意义($P>0.05$),而旋转极限位同侧椎动脉流速,管径均增加,屈曲旋转极限位同侧仅管径增加,差异有统计学意义($P<0.05$),流量差异无统计学意义($P>0.05$)。结论:颈部不同的旋转相关极限体位对椎动脉血流产生不同的影响,后伸旋转极限位可能增加椎基底动脉血流量。

［关键词］ 颈部;极限旋转体位;椎动脉血流

［中图分类号］ R681.5 ［文献标志码］ A ［文章编号］1005-0205(2017)03-0017-04

Effect of Cervical Rotation-related Limit Position on Vertebral Artery Blood Flow

ZHANG Fayao[1,2△] *ZHAO Shusen*[1] *ZHU Liguo*[2]

[1] Qiqihar Hospital of Traditional Chinese Medicine, Qiqihar 161005, Heilongjiang China;

[2] Wangjing Hospital of China Academy of Chinese Medical Sciences, Beijing 100102, China.

Abstract **Objective**: To evaluate the effect of cervical rotation-related limit position on vertebral artery blood flow by using transcranial Doppler(TCD). **Methods**: Transcranial Doppler was used to measure blood flow velocity and artery diameter in the vertebral arteries of 40 healthy adults aged 19 to 21 years old(mean 20.2 years old) in the positions as follows: neck neutral position, ipsilateral rotation limit, ipsilateral flexion rotational limit, ipsilateral extension of rotation limit, contralateral rotation limit, contralateral flexion rotation limit, contralateral extension rotation limit, supine, supine ipsilateral rotation limit, supine contralateral rotation limit and so on. The vertebral artery in the cervical 3-4 segment were measured, the blood flow and the total blood flow were obtained by calculation. The total blood flow was the sum of bilateral vertebral artery flow under the same position. The data including the vertebral artery flow rate, diameter, flow and the activity of neck angle were statistically analyzed. **Results**: The total blood flow in posterior rotation limit position was higher than that in the rotation limit position, flexion rotation limit position and supine rotation limit position($P<0.05$), and there was no significant difference between the three positions($P>0.05$). Compared with the neutral position, the contralateral vertebral artery blood flow were reduced in the rotational limit position and flexion limit ($P<0.05$), and there was no significant difference between the two group($P>0.05$), while the flow velocity and the diameter of ipsilateral vertebral artery in rotation limit position were both increased, for the ipsilateral vertebral artery in flexion rotation limit position, only its diameter was increased($P<0.05$), there was no significant difference in the flow velocity($P>0.05$). **Conclusion**: The different rotation-related limit positions of the neck have different effects on the blood flow of the vertebral arteries, and the posterior rotation limit can increase the vertebrobasilar blood flow.

Keywords: neck; limit rotation positions; vertebral artery blood flow

[1] 黑龙江齐齐哈尔市中医医院(黑龙江 齐齐哈尔,161005)

[2] 中国中医科学院望京医院

△通信作者 E-mail:jipinningmeng@126.com

?1994-2017 China Academic Journal Electronic Publishing House. All rights reserved. http://www.cnki.net

日常生活中颈椎运动往往是多方向的耦合运动。颈椎旋转体位与椎动脉的关系是诸如常发生在颈椎屈伸及旋转时的颈性眩晕，转颈试验检查，颈椎旋提手法治疗等临床诊疗的基础研究内容。经颅彩多普超声检查(TCD)是目前诊断椎动脉形态、血液动力学指标的常用方法，操作简便，准确率高。目前研究多限于颈椎有限旋转体位，临床研究对象多为有颈性眩晕患者，或者做三维有限元模型的构建分析，并不能客观反映生理状态下耦合旋转体位与椎动脉血流的关系。本研究旨在通过 TCD 对生理条件立位和卧位状态颈椎各种旋转相关极限体位下，椎动脉管径、血流速、血流量等的比较，探讨不同旋转体位对椎动脉血流的影响，为其深入研究提供参考，现报告如下。

1 研究对象和方法

1.1 研究对象

选取 40 例年轻健康受试者：来自齐齐哈尔市某普通高校；年龄 19～21 岁，平均 20.2 岁；其中男 20 例，女 20 例。纳入标准：身体健康，无内科相关疾病；无颈部疾病史；近 3 个月无颈部不适症状；颈椎正侧位 X 线证实无颈椎形态、曲度异常者；同意参与研究者；经医院伦理委员会审核批准。排除标准：经椎动脉多普勒超声检查有血管缺失、闭塞、斑块形成等异常者；有神经系统或精神类疾病者；受试过程中出现眩晕、头痛及其他不适症状者。

1.2 测量方法

采用飞利浦公司 IU22 型经颅多普勒超声诊断仪，使用 2 MHz 脉冲多普勒探头，一名经验丰富的医生操作，检测方法及标准参照焦明德[1]主编的《实用经颅多普勒超声学》。受试者取坐位，常规头部中立位，检测颈椎 3/4 节段椎动脉收缩期峰值的血流速度(V_p)，椎动脉内径(D)；再令受试者行对侧旋转至极限，稳定 5 s 后，测量 V_p 及 D 值；再次回正头部，做屈曲最大后，缓慢对侧旋转极限稳定 5 s 后测量；同理，回正头部后，后伸颈部至最大后，再次缓慢对侧旋转极限稳定后 5 s 后测量；同理行同侧旋转，屈曲同侧旋转，后伸同侧旋转的测量。再次行对侧椎动脉的 7 个体位的测量。休息 1 min 后，患者去枕平卧，行椎动脉常规体位，同侧极限旋转位，对侧极限旋转位 V_p 和 D 值的测量。所有测量节段均位于颈椎 3/4 节段椎动脉。

1.3 统计学方法

本研究对 40 个受试者 80 条椎动脉分别在坐位下中立位、同侧极限旋转、同侧极限屈曲旋转、同侧极限后伸旋转、对侧极限旋转、对侧极限屈曲旋转、对侧极限后伸旋转等和卧位下常规体位、同侧极限旋转、对侧极限旋转等共 10 个体位血流速度(V_p)，椎动脉内径(D)，血流量(Q)进行数据分析(见表 1)。旋转位总血流量($QZ_{立旋}$)为同一受试者立位最大旋转同侧血管血流量＋旋转对侧血管血流量；卧位旋转位总血流量($QZ_{卧旋}$)为同一受试者卧位最大旋转同侧血管血流量＋旋转对侧血管血流量；屈曲旋转位总血流量($QZ_{曲旋}$)为同一受试者最大屈曲旋转同侧血管血流量＋屈曲旋转对侧血管血流量；后伸旋转位总血流量($QZ_{伸旋}$)为同一受试者最大后伸后伸同侧血管血流量＋后伸旋转对侧血管血流量，使用 SPSS 22.0 软件随机区组设计的方差分析，组间两两比较进行 LSD-t 检验或者 SNK-q 检验。

$$Q=V_p\times(D/2)2\pi$$

表 1 各体位椎动脉血管及血流参数值比较($\bar{x}\pm s$)

体位	流速(cm/s)	管径直径(mm)	血流量(mL/s)
中立位	56.22±3.67	3.10±0.08	4.34±2.38
对侧旋	49.13±2.51	2.89±0.08	3.31±1.36
屈曲对侧旋	45.36±3.53	2.98±0.07	3.09±1.25
后伸对侧旋	57.04±2.57	2.92±0.08	4.10±1.78
同侧旋	64.72±2.76	3.40±0.08	5.36±2.19
屈曲同侧旋	53.80±3.04	3.46±0.08	4.90±2.16
后伸同侧旋	67.41±2.45	3.45±0.08	6.48±2.89
卧位常规	53.87±2.32	3.20±0.09	4.35±1.97
卧位对侧旋	52.04±2.01	3.06±0.08	3.62±1.64
卧位同侧旋	50.76±3.08	3.24±0.08	4.29±1.89
F	16.36	24.58	19.66
P	<0.01	<0.01	<0.01

2 结果

80 条椎动脉通畅性完好，无斑块形成及缺如，在测量过程中未发生受试者头晕、恶心、颈部疼痛、上肢麻木等症状。对各体位活动角度进行测量，结果如下：旋转角度为 63°±6.2°；屈曲旋转(极限)为旋转 45°±3.1°，屈曲 36°±5.7°；后伸旋转(极限)为旋转 33°±6.1°，后伸 34°±4.7°.

椎动脉在立位同侧旋转极限位较平卧同侧旋转体位，血流速和血流量高，差异有统计学意义($P<0.05$)(表 2，表 3)。立位：1)椎动脉在对侧极限旋转和极限屈曲对侧旋转体位时较中立位流速和血流量均有减少流速，差异有统计学意义($P<0.05$)，而二者血流量、血管管径差异无统计学意义($P>0.05$)(表 2～4)；2)与中立位相比，极限旋转同侧血流量增加，差异有统计

?1994-2017 China Academic Journal Electronic Publishing House. All rights reserved. http://www.cnki.net

学意义($P<0.05$)，极限屈曲同侧旋转血流量无明显增加，差异无统计学意义($P>0.05$)；同侧极限屈曲旋转和同侧极限后伸旋转血管管径增宽大于单纯同侧极限旋转，差异有统计学意义($P<0.05$)(表 2～4)；3)立位同侧极限旋转较卧位同侧极限旋转椎动脉血流量增加，差异有统计学意义($P<0.05$)(表 3)；4)极限后伸旋转位总血流量大于极限屈曲旋转，单纯极限旋转体位，及卧位极限旋转体位，差异有统计学意义($P<0.05$)，而后三者差异无统计学意义($P>0.05$)(表 5，6)。d 为均数差值，Sx 为标准误。

表 2　各体位组间椎动脉流速比较

体位两两比较	均数差值(d)	标准误($S\bar{x}$)	P
中立位-对侧旋	8.67	2.66	<0.01
中立位-屈曲对侧旋	12.63	2.66	<0.01
对侧旋-屈曲对侧旋	3.74	2.66	0.38
同侧旋-卧位同侧旋	14.05	2.66	<0.01

表 3　各体位组间流量比较

体位两两比较	均数差值(d)	标准误($S\bar{x}$)	P
中立位-对侧旋	0.89	0.32	<0.01
中立位-屈曲对侧旋	1.20	0.32	<0.01
中立位-同侧旋	－1.21	0.32	<0.01
中立位-屈曲同侧旋	0.63	0.32	0.05
对侧旋-屈曲对侧旋	－0.25	0.32	0.53
同侧旋-卧位同侧旋	1.20	0.32	<0.01

表 4　各体位组间动脉管径比较

体位两两比较	均数差值(d)	标准误($S\bar{x}$)	P
对侧旋-屈曲对侧旋	0.01	0.05	0.77
同侧旋-屈曲同侧旋	－0.12	0.05	0.03
同侧旋-后伸同侧旋	－0.14	0.05	0.01

表 5　各体位总血流量 QZ(mL/s)

体位	血流量($\bar{x}\pm s$)
立位旋转总血流量($QZ_{立旋}$)	8.86±2.50
屈曲旋转位总血流量($QZ_{曲旋}$)	7.79±2.42
后伸旋转位总血流量($QZ_{伸旋}$)	10.39±3.38
卧位旋转总血流量($QZ_{卧旋}$)	7.84±2.50
F	13.08
P	<0.01

表 6　各体位总血流量组间比较

体位两两比较	均数差值(d)	标准误($S\bar{x}$)	P
立位旋转-屈曲旋转位	0.83	0.45	0.11
立位旋转-后伸旋转位	－1.80	0.45	<0.01
立位旋转-卧位旋转	0.69	0.45	0.11
屈曲旋转位-后伸旋转位	－2.64	0.45	<0.01
屈曲旋转位-卧位旋转	0.00	0.45	0.99
后伸旋转位-卧位旋转	－2.54	0.45	<0.01

3　讨论

颈性眩晕是临床好发病，旋转极限体位是研究颈性眩晕发病机制、诊断、治疗等各个环节的重要内容。首先，多由于椎基底动脉供血不足引起，归结原因主要有：1)椎动脉受在横突孔中受椎间孔狭窄，增生骨赘等直接压迫，引起椎动脉受压、牵张，过度迂曲等导致；2)颈部活动刺激交感神经纤维，其支配的椎动脉血管痉挛等[2]两种观点。临床中眩晕甚至猝倒与颈椎旋转动作有相关性，有研究表明旋转体位可以引起椎基底动脉供血不足，或者症状加重，有研究发现随着转颈角度的加大，尤其在转颈至 45°时，椎动脉血流下降最为显著，诱发眩晕占 80%[3]，Machaly 等[4]通过椎动脉超声检测颈部正位与旋转 60°时的血流速发现，颈椎退变组和眩晕组患者头部旋转时，对侧椎动脉流速下降更加明显，甚至导致眩晕发作[4]。颈椎疾病的正骨手法疗效确切[5-7]，操作过程中也多有颈椎旋转相关动作，旋转手法可以纠正椎间小关节紊乱，对深部的关节囊、椎间韧带、滑膜起到松解的作用[8]，也可以松解交感神经根袖处的某些粘连，解除交感神经受到异常刺激而引起的椎动脉痉挛[9]。因此，研究中以颈椎各种极限旋转体位下的椎动脉管径、血流速、流量等为主要研究对象，另外，以往研究中受试者为颈椎疾病患者[10-13]，体位对椎动脉血流影响的研究可能受到患者血管本身痉挛，骨赘压迫或恐惧疼痛、眩晕等不适感等干扰。本研究纳入健康青年人，受试过程中均未发生不适症状，依从性好，能更客观地反映体位对健康人椎动脉的影响。

通过本研究发现在颈椎后伸旋转体位时，椎动脉总血流量是大于卧位旋转，屈曲旋转及立位旋转的，因此在由于椎动脉引起的椎基底动脉供血不足的病人中，限制性适当后伸位可能对改善椎动脉供血和避免突发一过性脑缺血发作有预防作用。根据 2010 年《眩晕治疗专家共识》颈性眩晕至少应有以下特征：头晕或眩晕多出现在颈部活动后；部分患者颈扭转试验阳性[14]。本研究显示：与中立位比较，在极限旋转和极限屈曲旋转体位时对侧椎动脉血流均有减少，二者无差异，而同侧椎动脉旋转体位下流速、管径血流量均增加，同侧屈曲旋转体位下仅管径增加，血流量差异并无统计学意义，那么在旋转同侧供血不足的情况，屈曲旋转较单纯旋转可能更易引起眩晕症状加重，进而提高转颈试验的阳性率。关于立位极限旋颈试验和卧位极限旋颈试验的文献报道，诸兴明等[2]在通过对颈性眩晕患者和非颈性眩晕患者均进行作为旋颈试验后测量椎动脉流速，TCD 颈性眩晕组阳性率 80.60%，非颈性眩晕组 4.55%，认为转颈试验有助于颈性眩晕的诊断。刘铁军等[15]在俯卧位对 87 例血流速度变化不明显者再行转颈试验后有 65 例血流速度表现异常，使得总阳性率提高到 93%[15].本研究在仰卧位和中立位进行椎动脉总流量的分析，流量差异无统计学意义，而立

?1994-2017 China Academic Journal Electronic Publishing House. All rights reserved.　http://www.cnki.net

位同侧旋转血流量大于卧位同侧旋转，由于对基底动脉供血仍存在压力差，并不能推断卧位转颈试验较立位转颈试验阳性率更高。Thomas 等[16]研究认为，健康成人的脑血供系统能通过其他动脉血流的增加来弥补一条或多条血管血流量的减少，维持极限旋转体位因此也能反映整个脑脉管系统的代偿能力，而不仅仅是单独的椎基底动脉系统。头颈部屈曲旋转，后伸旋转等体位下椎动脉的临床报道较少，各旋转耦合体位下总血流量的测量困难，本研究表明颈部旋转极限相关体位对椎动脉管径、血流速、流量等造成不同的影响，对于颈部血管总血流计算方法，提高转颈试验阳性率等的深入研究有借鉴意义，仍需大样本量加以证实。

参考文献

[1] 焦明德. 实用经颅多普勒超声学[M]. 北京：北京医科大学协和医科大学联合出版社，1995：116.

[2] 诸兴明，黄丽娜，万琦，等. 经颅多普勒超声转颈试验在颈性眩晕诊断中的应用价值[J]. 脊柱外科杂志，2014，12(3)：173-175.

[3] 郄晓红，王海音. 经颅多普勒转颈试验对椎动脉血流的影响[J]. 空军总医院学报，2010，26(2)：82-83.

[4] Machaly SA，Senna MK，Sadek AG. Vertigo is associated with advanced degenerative changes in patients with cervical spondylosis CIl[J]. Clin Rheumatol，2011，30(12)：1527-1534.

[5] 王辉昊，詹红生，吕桦，等. 矫正颈椎"筋出槽骨错缝"手法治疗颈性眩晕的远期疗效观察[J]. 上海中医药杂志，2014，48(2)：51-55.

[6] 席智杰，梁倩倩，施杞. 有关颈椎定点旋转手法的几点思考[J]. 中国中医骨伤科杂志，2015，23(2)：65-67.

[7] 孙金水，李云. 龙氏正骨手法治疗颈椎小关节病错位型颈椎病的临床疗效观察[J]. 当代医学，2014，24(359)：35-36.

[8] 谢利民，张涛. 张氏手法治疗椎动脉型颈椎病疗效观察[J]. 中医正骨，1999，11(7)：411.

[9] 李义凯，钟世镇. 旋转手法对椎管内结构和容积影响的研究[J]. 中国中医骨伤科杂志，1997，5(6)：4-6.

[10] 赵美玲. 转颈试验对椎动脉型颈椎病患者椎-基底动脉血流速的影响[J]. 医学影像学杂志，2008，18(7)：724-726.

[11] 贾杰，闻春艳，张文杰. 转颈试验在彩色多普勒超声检测颅外段椎动脉诊断椎动脉型颈椎病的价值[J]. 中国实验诊断学，2015，19(9)：1544-1545.

[12] 李泽宇，博 林，王智光，等. 转颈屈颈及仰头试验与经颅多普勒和核磁共振血管成像评估椎动脉狭窄性眩晕的效应比较[J]. 中国临床康复，2005，9(18)：254-255.

[13] 叶洁，顾小华，许金海，等. 颈性眩晕的中医证型与脑血流动力学的相关性研究[J]. 中国中医骨伤科杂志，2014，22(3)：7-11.

[14] 何及，樊东升，孙宇. 颈性眩晕[J]. 中国实用内科杂志，2011，31(6)：414-415.

[15] 刘铁军，张西平. TCD转颈试验在诊断椎基底动脉供血不足中的应用价值[J]. 陕西医学杂志，2011，40(9)：1255-1256.

[16] Thomas LC，McLeod LR，Osmotherly PG，et al. The effect of end-range cervical rotation on vertebral and internal carotid arterial blood flow and cerebral inflow：A sub analysis of an MRI study[J]. Man Ther，2015，20(3)：475-480.

（收稿日期：2016-09-06）

（上接第16页）

参考文献

[1] 周学龙. 寰枢关节半脱位的结构因素及其伴发症状概述[J]. 医学综述，2008，14(18)：2794-2796.

[2] 林红雨，王琦，刘桂芳. 寰枢关节半脱位的影像诊断及临床应用[J]. 齐鲁医学杂志，2014，29(6)：562-564.

[3] 谢再明，王兆杰. 寰枢关节半脱位临床诊断的研究进展[J]. 安徽卫生职业技术学院学报，2014，13(6)：25-26.

[4] 齐伟，王朝辉，王之虹. 寰枢关节半脱位诊断标准研究[J]. 长春中医药大学学报，2012，28(4)：638.

[5] 鲍铁周，李新生，李志强，等. 诊断寰枢关节半脱位的一种新方法[J]. 中医正骨，2013，25(4)：66-68.

[6] 杨子明. 寰枢关节是否存在半脱位及其相关问题[J]. 中华外科杂志，2006，44(20)：1369-1375.

[7] 国家中医药管理局. 中医病证诊断疗效标准[S]. 南京：南京大学出版社，1994：23.

[8] 孙传兴. 临床疾病诊断依据治愈好转标准[M]. 2版. 北京：人民军医出版社，2002：511.

[9] Debemardi A，D Aliberti G，Talamonti G，et al. Thecraniovertebral junction area and the role of the ligaments and membranes[J]. Neurossurgery，2011，68(2)：291-301.

[10] 韩应超，潘杰，王善金，等. 上颈椎韧带对寰枢椎稳定性影响的生物力学研究[J]. 中华骨科杂志，2013，33(6)：628-634.

[11] 黄一琳，侯晓桦. 寰枢关节半脱位98例临床分析[J]. 颈腰痛杂志，2000，21(1)：43-44.

[12] 李生泉. 寰枢关节失稳80例临床影像表现分析[J]. 实用医技杂志，2009，16(7)：544.

[13] 宋涛，童斌斌. CT检查寰枢关节周围关系在寰枢关节半脱位中的诊断价值[J]. 中国乡村医药，2015，22(22)：61-62.

[14] 谢再明，王兆杰，何建垣. FSE PDWI 联合 T2WI STIR 在寰枢关节半脱位诊断中的应用[J]. 西部医学，2015，27(2)：212-214.

[15] 侯玉亭，刘辉，黄范利. 环枢关节侧方滑脱的X线诊断[J]. 中国煤炭工业医学杂志，2001，4(10)：838-838.

[16] 李晶，陈禾丽，吴望一. 颈椎牵引的力学实验与临床运用. 中华理疗杂志，1992，15(3)：133.

[17] 杨利学，刘智斌，祝海滨. 颈椎病角度牵引研究近况[J]. 中国中医骨伤科杂志，2006，14(6)：82-84.

[18] 朱海波，贾连顺，寇庚，等. 枢椎解剖学测量及临床意义[J]. 解剖学杂志，1997(4)：305-309.

[19] 胡勇，杨述华，谢辉，等. 人工寰齿关节设计依据及可行性分析[J]. 中国骨伤，2007，20(9)：587-591.

[20] 徐军. 脊柱牵引治疗技术(续三)：颈椎牵引技术[J]. 中国临床康复，2002，6(6)：778-781.

[21] 李勇，张泽胜，王伶俐，等. 不同牵引角度治疗颈椎病的三维有限元分析研究[J]. 新中医，2008，40(9)：63-64.

（收稿日期：2016-09-11）

?1994-2017 China Academic Journal Electronic Publishing House. All rights reserved. http://www.cnki.net
