# Supplementary material for: Kinematics Analysis of Cervical Rotation-Traction Manipulation Measured by a Motion Capture System
Source: Evid Based Complement Alternat Med. 2017 Oct 11;2017:5293916. doi: 10.1155/2017/5293916 (PMC5660796; doi:10.1155/2017/5293916)
Supplement: Supplementary file 1 — Procedure of CRTM, the related result of CRTM, the related reference. [file 5293916.f1.zip › Supplementary Material/Appendix 7.pdf]

·临床研究·

旋提手法治疗神经根型颈椎病的疗效观察

王乾，朱立国，高景华，于杰，
冯敏山，杨克新，王尚全，罗杰，张清，张威

（中国中医科学院望京医院，北京　100102）

摘　要　目的：观察旋提手法治疗神经根型颈椎病的临床疗效。方法：将 110例患者随机分为两组，治疗组采用旋提手法治疗 2周，对照组采用牵引疗法治疗 2周。结果：旋提手法治疗神经根型颈椎病临床疗效经统计学分析效果明显优于对照组。结论：旋提手法治疗神经根型颈椎病临床疗效显著，手法操作安全，值得临床推广。

关键词　神经根型颈椎病　治疗　推拿　旋提手法　临床研究

Clinical observation on rotation–traction manipulation for treatment of the cervical spondylotic radiculopathy

WANG Qian*, ZHU Li-guo, GAO Jing-hua, YU Jie, FENG Ming-shan, YANG Ke-xin, WANG Shang-quan, LUO Jie, ZHANG Qing, ZHANG Wei

Abstract　Objective: to study therapeutic effects of rotation–traction manipulation for the treatment of the cervical spondylotic radiculopathy. Methods: 110 patients were randomly divided into two groups. The patients in the treatment group were treated with rotation–traction manipulation, and the patients in the control group were treated with traction. Results: The therapeutic effects of treatment group were obviously better than those of control group by statistical evaluation. Conclution: It is obvious for the therapeutic effects of rotation–traction manipulation, which is safe and worthy of practicing.

Key words　the cervical spondylotic radiculopathy; treatment; massage therapy; rotation-traction manipulation; clinical research

随着社会发展，生活节奏的加快，以及长时间伏案工作，电脑的普及，都使人长时间保持单一姿势，容易导致肌肉韧带疲劳，致使颈椎病的发病率正在逐年提升，已逐渐成为骨科疾病发病率之首，而神经根型颈椎病在颈椎病发病中占 50% ~60%[1]。旋转手法用以治疗神经根型颈椎病已有悠久的历史，并且得到广大骨科专家的认可。经过长期实践探索，我们在该手法基础上，加以创新，总结出了颈椎旋提手法，临床上用于治疗神经根型颈椎病，效果良好。为进一步验证其疗效，自 2004年 10月 ~2006年 8月，我们将收治的 110例神经根型颈椎病人，随机分为两组，以常规的牵引疗法为对照，进行了系统的对照观察研究，报告如下。

1　临床资料

共纳入 110例病人，均符合 1992年全国第二届颈椎病专题座谈会颈椎病的诊断标准[2]。采用查随机数字表方法随机分为两组，手法组 54例，男 30例，女 24例，牵引组 56例，男 29例，女 27例，两组性别比较，经 χ^2 检验，$\chi^2=0.267$，$P>0.05$ 无统计学意义；手法组年龄 45 ~65岁，平均 51.5岁，牵引组年龄 45 ~63岁，平均 53.2岁，两组年龄比较，经 χ^2 检验，$\chi^2=0.278$，$P>0.05$ 无统计学意义；手法组病程 0.3 ~12年，平均 4.6年，牵引组病程 0.1 ~16年，平均 3.9年，两组患者病程比较，经 χ^2 检验，$\chi^2=1.536$，$P>0.05$ 无统计学意义；两组症状体征积分，手法组（28.32±3.72）分，牵引组（27.89±3.01）分，经 t检验，$t=0.766$，$P>0.05$ 无统计学意义，两组比较具有可比性。

2　病例选择标准

2.1　纳入标准　① 符合根性颈椎病诊断标准；② 年龄 45 ~65岁；③ 病程小于（或等于）5年；④ 如果已经接受过其他手法或非手术治疗，经过 5 d以上的洗脱期。

2.2　排除标准　①颈椎外病变（如胸廓出口综合征、腕管综合征、肩周炎）；②疑有或已确诊的颈椎及椎管内肿瘤者，脑部肿瘤者；③有出血倾向的血液病患者；④ 骨关节结核、骨髓炎及老年性骨质疏松症等；⑤ 诊断不明确的脊柱损伤伴脊髓损伤症状者；⑥发育性椎

通讯作者：朱立国　Tel：84739194　Email：zhlg95@yahoo.com.cn

?1994-2015 China Academic Journal Electronic Publishing House. All rights reserved.　http://www.cnki.net

管狭窄者(椎管比值=椎管矢状径/椎体矢状径<0.75);⑦严重的心、肺、脑疾病患者;⑧手法部位有严重皮肤损伤或皮肤病者;⑨脊髓型颈椎病;⑩不愿意接受研究者。

3 治疗方法

3.1 旋提手法[3]　病人端坐位,颈部自然放松,医者采用按法、揉法、滚法等手法放松颈部软组织,3~5 min;让患者的头部水平旋提至极限角度,最大屈曲,达到有固定感;医生以肘部托患者下颌,轻轻向上牵引 3~5 s;嘱其放松肌肉,肘部用短力快速向上提拉;操作成功可以听到一声或多声弹响。

3.2 牵引疗法[4]　患者坐位,带枕颌布兜牵引,头部向前微屈,约前倾 10~15°,并以病人感觉舒适且能减轻症状为准;牵引重量从 3 kg开始,按 0.5 kg标准逐渐增加,最大不超过 6 kg。每次牵引 30 min。

3.3 治疗方案　手法组采用旋提手法治疗 2周,隔天 1次,共 7次。牵引组采用牵引疗法治疗,连续治疗 2周,每天 1次,共 14次。症状消失可以停止治疗。分别于治疗后第 1、3、5、7、9、11、13 d及治疗后 1个月 8个时间点进行观察,记录观察指标。

4 疗效观察

4.1 观察指标　①一般项目:包括姓名、性别、年龄、病程、病史等。②主要症状体征:主要症状体征积分采用神经根型颈椎病 35分积分表[5],主要观察指标包括:颈臂疼痛、麻木、压痛、颈部活动度、肌力等。

4.2 评定标准　临床控制:症状体征消失或基本消失,疗效指数≥90%;显效:症状体征明显改善,疗效指数≥70%,<90%;有效:症状体征均有好转,疗效指数达≥30%,<70%;无效:症状体征无明显改善,疗效指数达 30%以下。

4.3 统计方法　统计分析采用 SPSS统计分析软件进行计算。

5 治疗结果

5.1 疗效比较　经治疗按上述标准评定,手法组总有效率为 94.4%;牵引组总有效率为 85%。手法组与牵引组之间总体疗效比较,经 Ridit分析,U=2.673,P<0.01,差异有统计学意义,说明手法组疗效优于牵引组(见表 1)。

5.2 治疗前后症状体征总积分比较　两组治疗结束后,手法组与牵引组症状体征积分比较,经 t检验,手法组 t=12.63,P<0.01,牵引组 t=10.43,P<0.01,差异均有统计学意义,说明手法和牵引治疗本病,均可以明显改善症状体征;手法组与牵引组治疗前后症状体征积分差值比较,经 t检验分析,t=4.887,P<0.01,差异有统计学意义,说明手法改善症状体征的作用优于牵引疗法(见表 2)。

表 1　两组疗效比较

组　别	例数	临床控制	显效	有效	无效
手法组	54	15	24	13	3
牵引组	56	4	16	28	8
合　计	110	19	40	41	11

表 2　治疗前后症状体征积分比较 ($\bar{x}\pm s$)

组　别	例数	治疗前	治疗后	差值
手法组	54	28.32±3.72	5.89±1.89	14.64±5.01
牵引组	56	27.89±3.01	8.99±2.21	17.89±5.43

5.3 不良反应　两组均未出现不良反应。

5.4 主要指标比较

5.4.1 两组治疗前后疼痛积分比较　两组治疗前后疼痛积分比较,经 t检验,手法组 t=10.13,P<0.01,牵引组 t=8.43,P<0.01,差异均有统计学意义,说明手法和牵引治疗本病,均可明显减轻疼痛症状。手法组与牵引组治疗前后疼痛积分差值比较,经 t检验分析,t=3.58,P<0.01,差异有统计学意义,说明手法改善疼痛的作用优于牵引疗法(见表 3)。

表 3　两组治疗前后疼痛积分比较 ($\bar{x}\pm s$)

组　别	例数	治疗前	治疗后	差值
手法组	54	4.46±1.82	1.89±1.09	2.24±1.51
牵引组	56	4.51±1.51	2.95±1.21	1.35±1.03

5.4.2 两组治疗后压痛比较　两组治疗前后压痛积分比较,经 t检验,手法组 t=8.34,P<0.01,牵引组 t=6.92,P<0.01,差异有统计学意义,说明手法和牵引治疗本病,均可明显改善压痛。手法组与牵引组治疗前后压痛积分差值比较,经 t检验,t=2.12,P<0.05,差异有统计学意义,说明手法改善压痛的作用明显优于牵引疗法(见表 4)。

表 4　两组治疗前后压痛评分比较 ($\bar{x}\pm s$)

组　别	例数	治疗前	治疗后	差值
手法组	54	5.66±2.10	3.21±1.13	2.43±1.31
牵引组	56	5.72±1.89	4.35±1.42	1.44±1.05

5.4.3 两组治疗前后活动度比较　两组治疗前后活动度积分比较,经 t检验,手法组 t=8.34,P<0.01,牵引组 t=6.92,P<0.05,差异有统计学意义,说明手法和牵引治疗本病,均可明显改善活动度(见表 5)。

?1994-2015 China Academic Journal Electronic Publishing House. All rights reserved.　http://www.cnki.net

表 5 两组治疗前后活动度比较 $\bar{x}\pm s$

组　别	例数	治疗前	治疗后
手法组	54	2.12±0.90	0.87±0.45
牵引组	56	2.14±0.89	1.25±0.62

6 讨　论

6.1 神经根型颈椎病的病因病理　有以下三种学说:①机械压迫学说:Goldstein认为神经根可能受到来自前方的钩椎关节骨赘和来自后方的小关节骨赘的双重压迫。随着年龄的增长及退行性病变,黄韧带逐渐失去弹性,在颈后伸时会突向椎管,压迫脊髓或神经根。椎间盘向后内侧、中央旁侧突出会压迫脊髓,向后外侧、椎间孔内或外侧突出会压迫神经根[6]。②化学性神经根炎学说:化学物质和神经肽类等致痛物质直接激发疼痛和降低痛阈,损伤或炎症组织释放的内源性物质在伤害刺激和疼痛感受放电之间起着桥梁作用。③自身免疫学说:退变或突出的椎间盘发生免疫反应,许多神经多肽在免疫细胞的作用下释放,导致神经根型颈椎病根性痛的发病。

6.2 旋提手法治疗神经根型颈椎病作用机理　目前,旋提手法治疗颈椎病基础研究滞后于临床,机理研究较少,多为推测,有待进一步加强。现有的认识可概括为以下几个方面:①解除肌肉痉挛:临床触诊时可以摸到收缩变硬的肌肉或僵硬无弹性的条索状肌腹。除准备手法松解痉挛僵硬的颈肩肌群,快速推扳和旋提也可突然牵拉松解肌肉的高张力,使异常的肌肉张力恢复正常。②纠正关节错位:旋提手法可调整椎间盘与神经根的位置、调整钩锥关节、恢复正常的颈椎关节解剖序列,有利于组织水肿的消退、静脉的回流、神经根周围炎症的减退,达到治疗目的。③松解粘连:旋提手法可使神经根和关节周围的粘连得到一定程度的松解。有研究在伴有颈椎侧弯的新鲜尸体上直接观察旋提手法对颈椎侧弯时椎管结构发生的变化,旋提手法对颈椎管内解剖结构的改变,如椎管截面积、椎管矢状径、神经根袖等具有较明显的作用。旋提时,对侧神经根袖位移明显,这有助于解除神经根袖处的某些粘连。侧弯时,对侧神经根袖位移较明显。前屈旋提对下位神经根位移的影响较大,有利于松解神经根袖处和椎管内的某些粘连,从而达到治疗目的。④解除滑膜嵌顿:椎间小关节有独立的关节囊,关节囊内滑膜皱襞上有丰富的感觉神经纤维和P物质,当颈随头作各个方向的运动,椎间关节间隙增大时,关节囊内层的滑膜或滑膜皱襞就有可能嵌入,成为疼痛源。颈部的推扳或旋提手法可使嵌入的滑膜或滑膜皱襞得到解除,从而缓解疼痛。

本研究证实旋提手法治疗神经根型颈椎病具有显著疗效,较之牵引(牵引组)有一定优效性。牵引能拉开椎间隙,扩大椎间孔,解除上下关节突对神经根的机械性卡压,缓解神经根的压迫和刺激症状作用。本研究中也证实通过这些作用,手法组神经根型颈椎病症状体征积分改善优于牵引组。

随访中手法组患者,疗效巩固同时还有进一步恢复可能,也说明旋提手法能纠正颈椎动力性、静力性失衡的恶性循环,建立良性循环,有利于神经根型颈椎病进一步康复。由于条件限制本研究随访 4周时间较短,远期随访疗效有待进一步观察。

6.3 旋提手法治疗神经根型颈椎病的安全性　颈椎病的手法治疗只要方法正确,操作恰当,严格地选择适应证与禁忌症,不但疗效显著,而且也是安全可靠的。本研究中要求患者头部主动水平旋提至极限角度,最大屈曲,达到有固定感情况下向上提拉,使关节囊紧束,关节绞锁,充分发挥“筋束骨”的功能,调整关节及局部微细结构。较之患者被动情况下由医者在关节囊松弛下旋提,本法不容易超出生理极限,提高疗效同时增加可控性,避免对局部组织的不良刺激,也是本研究中未出现不良反应的主要原因之一。总之,本研究中的旋提手法是治疗神经根型颈椎病安全且有效的方法,值得临床推广。

7 参考文献

[1] 朱立国,于杰,高景华.旋转手法治疗神经根型颈椎病研究概述[J].中医正骨,2005,17(8):69-70.

[2] 孙宇.第二届颈椎病专题座谈会纪要[J].中华外科杂志,1993,31(8):472-476.

[3] 朱立国,于杰,高景华,等.旋转手法治疗神经根型颈椎病对疼痛的 VAS评分临床研究[J].北京中医,2005,24(5):297-298.

[4] 杨克勤.脊柱疾患的临床与研究[M].北京:北京出版社,1993:539.

[5] 朱立国,张清,高景华,等.旋转手法治疗神经根型颈椎病的临床疗效研究[J].中国骨伤,2005,18(8):489-490.

[6] Goldstein B. Anatomic issues related to cervical and lumbosacral radiculopathy. Phys Med Rehabil Clin N Am, 2002, 13:423-437.

(2008-12-15收稿　2009-03-06修回)

?1994-2015 China Academic Journal Electronic Publishing House. All rights reserved.　http://www.cnki.net
