# Supplementary figures and images for: Kinematics Analysis of Cervical Rotation-Traction Manipulation Measured by a Motion Capture System
Source: Evid Based Complement Alternat Med. 2017 Oct 11;2017:5293916. doi: 10.1155/2017/5293916 (PMC5660796; doi:10.1155/2017/5293916)

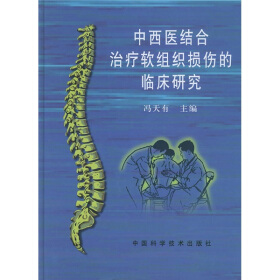

Supplement: Supplementary file 1 — Procedure of CRTM, the related result of CRTM, the related reference. [file 5293916.f1.zip › Supplementary Material/Appendix 4 Feng Tianyou.jpg]
